# The Nuclear Immune Receptor *RPS4* Is Required for *RRS1^SLH1^*-Dependent Constitutive Defense Activation in *Arabidopsis thaliana*

**DOI:** 10.1371/journal.pgen.1004655

**Published:** 2014-10-23

**Authors:** Kee Hoon Sohn, Cécile Segonzac, Ghanasyam Rallapalli, Panagiotis F. Sarris, Joo Yong Woo, Simon J. Williams, Toby E. Newman, Kyung Hee Paek, Bostjan Kobe, Jonathan D. G. Jones

**Affiliations:** 1 The Sainsbury Laboratory, Norwich Research Park, Norwich, United Kingdom; 2 Bioprotection Research Centre, Institute of Agriculture and Environment, Massey University, Palmerston North, New Zealand; 3 School of Life Sciences and Biotechnology, Korea University, Seoul, Republic of Korea; 4 School of Chemistry and Molecular Biosciences, Institute for Molecular Bioscience and Australian Infectious Diseases Research Centre, University of Queensland, Brisbane, Australia; The University of North Carolina at Chapel Hill, United States of America

## Abstract

Plant nucleotide-binding leucine-rich repeat (NB-LRR) disease resistance (R) proteins recognize specific “avirulent” pathogen effectors and activate immune responses. NB-LRR proteins structurally and functionally resemble mammalian Nod-like receptors (NLRs). How NB-LRR and NLR proteins activate defense is poorly understood. The divergently transcribed Arabidopsis *R* genes, *RPS4* (resistance to *Pseudomonas syringae* 4) and *RRS1* (resistance to *Ralstonia solanacearum* 1), function together to confer recognition of *Pseudomonas* AvrRps4 and *Ralstonia* PopP2. *RRS1* is the only known recessive NB-LRR *R* gene and encodes a WRKY DNA binding domain, prompting suggestions that it acts downstream of RPS4 for transcriptional activation of defense genes. We define here the early RRS1-dependent transcriptional changes upon delivery of PopP2 *via Pseudomonas* type III secretion. The Arabidopsis *slh1* (*sensitive to low humidity 1*) mutant encodes an RRS1 allele (RRS1^SLH1^) with a single amino acid (leucine) insertion in the WRKY DNA-binding domain. Its poor growth due to constitutive defense activation is rescued at higher temperature. Transcription profiling data indicate that RRS1^SLH1^-mediated defense activation overlaps substantially with AvrRps4- and PopP2-regulated responses. To better understand the genetic basis of RPS4/RRS1-dependent immunity, we performed a genetic screen to identify *suppressor of* slh1 *immunity* (*sushi*) mutants. We show that many *sushi* mutants carry mutations in *RPS4*, suggesting that RPS4 acts downstream or in a complex with RRS1. Interestingly, several mutations were identified in a domain C-terminal to the RPS4 LRR domain. Using an *Agrobacterium*-mediated transient assay system, we demonstrate that the P-loop motif of RPS4 but not of RRS1^SLH1^ is required for RRS1^SLH1^ function. We also recapitulate the dominant suppression of RRS1^SLH1^ defense activation by wild type RRS1 and show this suppression requires an intact RRS1 P-loop. These analyses of RRS1^SLH1^ shed new light on mechanisms by which NB-LRR protein pairs activate defense signaling, or are held inactive in the absence of a pathogen effector.

## Author Summary

How plant NB-LRR resistance proteins and the related mammalian Nod-like receptors (NLRs) activate defense is poorly understood. Plant and animal immune receptors can function in pairs. Two Arabidopsis nuclear immune receptors, RPS4 and RRS1, confer recognition of the unrelated bacterial effectors, AvrRps4 and PopP2, and activate defense. Using delivery of PopP2 into Arabidopsis leaf cells *via Pseudomonas* type III secretion, we define early transcriptional changes upon RPS4/RRS1-dependent PopP2 recognition. We show an auto-active allele of RRS1, RRS1^SLH1^, triggers transcriptional reprogramming of defense genes that are also reprogrammed by AvrRps4 or PopP2 in an RPS4/RRS1-dependent manner. To discover genetic requirements for RRS1^SLH1^ auto-activation, we conducted a suppressor screen. Many *suppressor of* slh1 *immunity* (*sushi*) mutants that are impaired in RRS1^SLH1^-mediated auto-activation carry loss-of-function mutations in RPS4. This suggests that RPS4 functions as a signaling component together with or downstream of RRS1-activated immunity, in contrast to earlier hypotheses, significantly advancing our understanding of how immune receptors activate defense in plants.

## Introduction

Plant innate immunity relies on two layers of pathogen detection. Cell surface-localized pattern recognition receptors detect pathogen-associated molecular patterns (PAMPs) of invading microorganisms and activate PAMP-triggered immunity (PTI) [1]. Successful pathogens must circumvent PTI to colonize plants, and many bacterial pathogens use type III secretion (T3S) to deliver effectors that suppress PTI into plant cells [1]. Effectors can be detected directly or indirectly by plant disease resistance (R) proteins, which then activate effector-triggered immunity (ETI) generally together with a hypersensitive response (HR) of the infected tissue [2]. Most intracellular R proteins are modular, with an amino-terminal coiled coil (CC) or Toll/interleukin-1 receptor/R protein (TIR) domain, a nucleotide binding (NB) domain and a leucine-rich repeat (LRR) domain [3]. Some NB-LRR proteins also carry an additional carboxyl-terminal extension, the function of which is unknown [3]. In addition, NB-LRR protein function generally requires an intact P-loop motif (GxxxxGKT/S) in the NB domain, presumably for ATP binding and energy-dependent conformational changes [3], [4]. Plant NB-LRR proteins and mammalian Nod-like receptors (NLRs) exhibit both structural and functional similarities [5].

Signaling following TIR-NB-LRR protein activation requires other key regulators such as Enhanced Disease Susceptibility 1 (EDS1), the EDS1-related proteins PAD4 and SAG101, and biosynthesis of the plant hormone salicylic acid (SA) for full immunity [6]. EDS1 was recently reported to interact with several NB-LRR proteins [7], [8]. Mis-regulation of R protein accumulation, localization or activation can cause constitutive defense responses, which are usually deleterious or lethal. For instance, the dwarf *suppressor of npr1-1, constitutive 1* (*snc1*) mutant carries a point mutation between NB and LRR domains of the TIR-NB-LRR protein SNC1, which results in constitutive defense signaling [9], [10]. Suppression of the stunted *snc1* phenotype in *mos* (*modifier of* snc1) mutants allowed the identification of several genes required for nuclear defense signaling [11]–[14].

Although most R proteins function to recognize a corresponding avirulent effector (Avr), some NB-LRR proteins appear to act downstream of R protein activation. The tobacco and tomato CC-NB-LRR proteins, “N-required gene 1” (NRG1), and “NB-LRR protein required for HR-associated cell death 1” (NRC1), are required for TIR-NB-LRR protein N-mediated resistance to tobacco mosaic virus and receptor-like protein Cf-4-mediated resistance to tomato leaf mold pathogen, respectively [15], [16]. Arabidopsis CC-NB-LRR Activated Disease Resistance 1 (ADR1) family proteins are required for SA-dependent ETI [17]. The Arabidopsis accession Col-0 downy mildew resistance locus *RPP2* comprises two distinct closely linked NB-LRR genes *RPP2A* and *RPP2B*, both of which are required for resistance [18]. The rice *Pia* locus for blast (*Magnaporthe*) resistance comprises two divergently transcribed CC-NB-LRR genes, RGA4 and RGA5, again both required for resistance [19]. In mammals, the NLR NAIP2 confers specific recognition of PrgJ, whereas NLRs NAIP5 and NAIP6 confer responses to flagellin. However, the NLR NLRC4 is required for defense responses to both PrgJ and flagellin [20], [21]. NLRC4 association with either NAIP2 or NAIP5/6, upon provision of PrgJ or flagellin respectively, is required for defense activation [20], [21].

The T3S effectors AvrRps4 and PopP2 from *Pseudomonas syringae* and *Ralstonia solanacearum* respectively, are recognized by paired TIR-NB-LRR proteins RPS4 (resistance to *P. syringae* 4) and RRS1-R (resistance to *R. solanacearum* 1), and activate ETI in Arabidopsis [22]–[24]. RRS1-R alleles, found in accessions Ws-2, No-0 and Nd-1, confer recognition of PopP2; the RRS1-S allele of Col-0 does not recognize PopP2, but does recognize AvrRps4 [22]–[24]. Lack of AvrRps4 recognition in accession RLD is due to non-synonymous mutations in RPS4, and RRS1-S in Col-0 is truncated compared to RRS1-R because of an early stop codon [24]–[26]. RPS4 and RRS1-R genetically function together, as plants lacking RPS4, RRS1-R or both show similar enhanced susceptibility to bacterial strains expressing AvrRps4 or PopP2 [25], [26]. RRS1 (also annotated as WRKY52) is an atypical NB-LRR protein that also carries a C-terminal WRKY DNA-binding domain [22].

In this study, we delivered PopP2 using *Pseudomonas* T3S by fusing it with the N-terminal region of AvrRps4 (AvrRps4N). *Pseudomonas*-delivered AvrRps4N:PopP2 triggers RPS4- and RRS1-dependent HR and immunity in resistant Arabidopsis genotypes when tagged with a nuclear localization signal (NLS) but not when tagged with a nuclear exclusion signal (NES). We show that the delivery of PopP2, or an inactive PopP2^C321A^ variant, from a *Pseudomonas fluorescens* strain (Pf0-1) that lacks other effectors [27], results in the induction of ETI-specific genes that overlaps substantially with previously reported AvrRps4-regulated genes [28], [29].

The presence of a single amino acid (Leu) insertion in the WRKY domain of RRS1-R (RRS1^SLH1^ hereafter) causes the recessive lethal phenotype of the *sensitive to low humidity 1* (*slh1*) mutant in No-0 [30]. RRS1^SLH1^-induced lethality is associated with enhanced defense gene expression and high SA accumulation. Similarly to other mutants displaying spontaneous cell death, *slh1* mutant growth can be restored to wild type phenotype at 28°C [30]–[32]. In contrast to *snc1*, the *slh1* mutant allele is recessive and heterozygotes show no constitutive defense activation [30]. *RRS1* is also recessive and an RRS1-R/RRS1-S heterozygote is unable to recognize PopP2 [22], [23].

Here, we used the conditional RRS1^SLH1^-mediated lethal phenotype to gain insights into RPS4/RRS1 gene pair function. Transcriptional profiling of the *slh1* mutant shows that genes induced during RRS1^SLH1^-mediated defense activation in the absence of Avr overlap with those induced by AvrRps4- or PopP2-triggered immunity. Genetic screening for mutations that suppress *slh1*-triggered aberrant immunity reveals the critical role of RPS4 in RRS1^SLH1^-mediated activation of defense signaling. Transient expression of RPS4 and RRS1^SLH1^ in tobacco results in HR in the absence of AvrRps4 or PopP2, which can be suppressed by co-expression of wild type RRS1-R, consistent with the recessive nature of RRS1^SLH1^. Our study sheds new light on how paired R proteins work cooperatively and illustrates the similarities between auto-active and Avr-dependent defense signaling.

## Results

### PopP2 triggers RPS4 and RRS1-dependent immune responses in Arabidopsis when delivered from *Pseudomonas* strains

**Figure 1:**
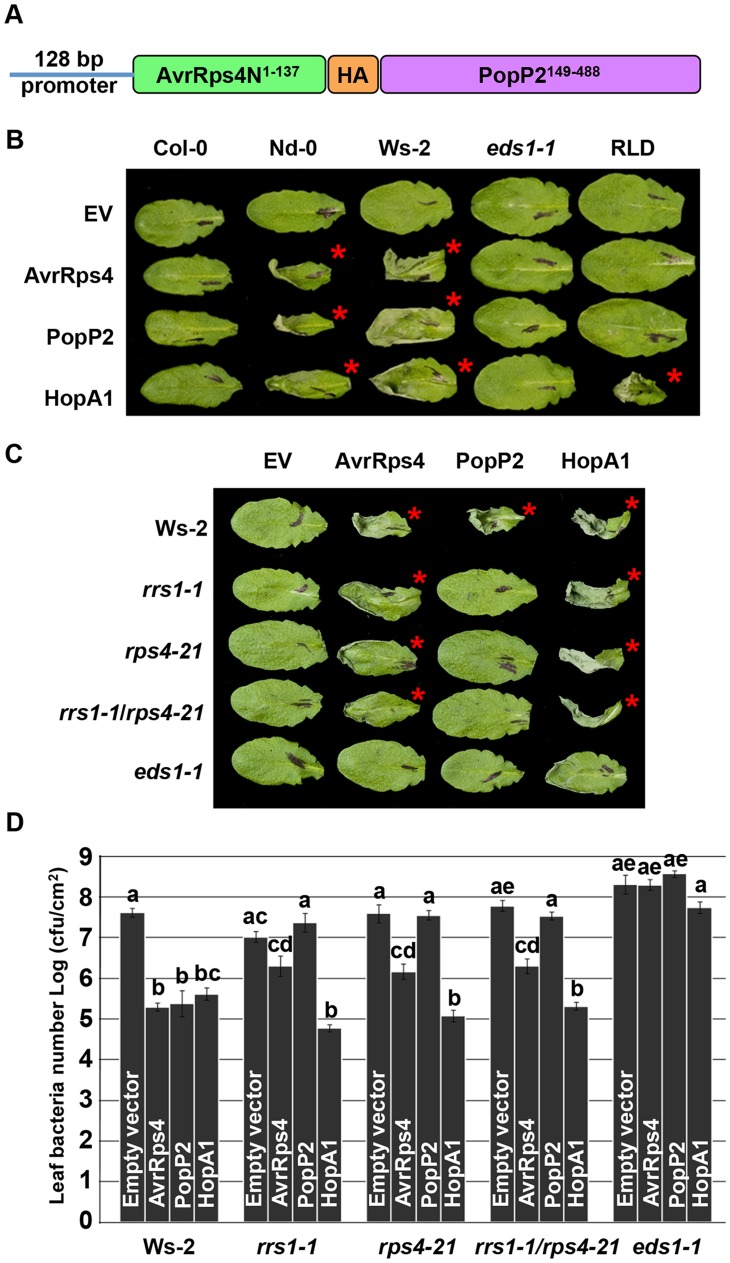
PopP2 triggers RPS4/RRS1/EDS1-dependent hypersensitive response and immunity when delivered from *Pseudomonas*. (*A*) AvrRps4N-PopP2 fusion construct. (*B*) *Pseudomonas fluorescens* Pf0-1(T3S)-delivered AvrRps4N:PopP2^149–488^ triggers an EDS1-dependent hypersensitive response (HR) in resistant Arabidopsis accessions. Five week-old Arabidopsis leaves were infiltrated with Pf0-1(T3S) strains expressing indicated avirulence proteins. Empty vector (EV) indicates AvrRps4N encoded by pEDV5 (see Figure S1). The photograph was taken at 24 hours post-infection (hpi). The red asterisks indicate the leaves showing HR. (*C*) Pf0-1(T3S)-delivered AvrRps4N:PopP2^149–488^ triggers an RPS4/RRS1-dependent HR in Ws-2 accession. (*D*) *Pseudomonas syringae* pv. *tomato* (*Pto*) DC3000-delivered AvrRps4N:PopP2^149–488^ triggers RPS4/RRS1-dependent immunity in accession Ws-2. Five week-old Arabidopsis leaves were infiltrated with *Pto* DC3000 strains and samples were taken at 4 dpi to recover bacteria from infected leaves. The results presented are the mean and standard error of the number of bacterial colonies recovered. Means labeled with the same letter are not statistically different at the 5% confidence level based on Tukey's test.

To compare AvrRps4- or PopP2-triggered HR and immunity, we established the delivery of PopP2 *via* the *Pseudomonas* T3S. We engineered pEDV6, a Gateway-compatible version of pEDV3 [33], to carry full-length or N-terminally truncated PopP2 variants (Figures 1A and S1A–B). pEDV6 enables expression of a translational fusion between the N-terminal part of AvrRps4 (137 first amino acids; hereafter, AvrRps4N) and an effector of interest. We used a non-pathogenic *Pseudomonas fluorescens* Pf0-1 engineered to carry a functional T3S system (hereafter, Pf0-1(T3S)) in HR assays because unlike *Pseudomonas syringae* pv. *tomato* (*Pto*) DC3000, Pf0-1(T3S) does not elicit non-specific tissue collapse. When delivered from Pf0-1(T3S) or *Pto* DC3000, PopP2^1–488^ (full-length) or PopP2^149–488^ triggered HR and immunity in Arabidopsis accession Ws-2, whereas the PopP2 variants that were further truncated did not (Figure S1C–D). Interestingly, the N-terminal 148 amino acids of PopP2 that include a nuclear localization signal (NLS) are dispensable in our assay. Based on this finding, we used the PopP2^149–488^ (hereafter, PopP2) variant for the rest of our experiments.

To verify that *Pseudomonas*-delivered PopP2 confers genotype-specific avirulence, we investigated the responses of Arabidopsis natural variants to PopP2. When delivered from Pf0-1(T3S), PopP2 and AvrRps4 triggered HR in accessions Nd-0 and Ws-2 whereas Col-0 and RLD showed no symptoms at 24 hours post-infection (hpi) (Figure 1B). Col-0 RRS1-S confers HR-deficient disease resistance to *Pst* DC3000 delivered AvrRps4 but not to PopP2 [22], [34]. In addition, transgenic expression of Ws-2 RRS1-R in Col-0 confers strong HR in response to *Pseudomonas*-delivered AvrRps4 [35]. HopA1 was used as an additional control; it triggers HR in Nd-0, Ws-2 and RLD, but not in Col-0, as expected. Next, we tested if Pf0-1(T3S)-delivered PopP2 triggers RPS4- and RRS1-dependent HR in Arabidopsis. Pf0-1(T3S)-delivered PopP2 triggered strong HR in wild type Ws-2 whereas Ws-2 *rrs1-1*, *rps4-21*, *rrs1-1*/*rps4-21* or *eds1-1* mutants did not show any response (Figure 1C). In contrast, Pf0-1(T3S)-delivered AvrRps4 triggered weak but robust HR even in the absence of RPS4 or RRS1 in Ws-2 (Figure 1C). When delivered from *Pto* DC3000, AvrRps4 triggered immunity in wild type Ws-2, *rrs1-1*, *rps4-21* or *rrs1-1*/*rps4-21* mutants because AvrRps4 recognition leads to *RPS4*/*RRS1*-dependent and -independent immunity (Figure 1D) [26]. To test if *Pseudomonas*-delivered PopP2 can trigger *RPS4/RRS1*-dependent immunity in Arabidopsis, we engineered a virulent *Pto* DC3000 to deliver PopP2. *Pto* DC3000 (PopP2) showed reduced virulence in wild type Ws-2 but not in *rrs1-1*, *rps4-21* or *rrs1-1*/*rps4-21* mutants compared to *Pto* DC3000 (pEDV5) indicating that *Pseudomonas*-delivered PopP2 triggers only RPS4/RRS1-dependent immunity (Figure 1D), consistent with previously reported *Ralstonia*-delivery assay results [26]. By contrast, HopA1-triggered immunity was not affected in *rrs1-1*, *rps4-21* or *rrs1-1*/*rps4-21* mutants compared with wild type Ws-2 (Figure 1D). All tested *Pto* DC3000 strains showed unrestricted growth in the *eds1-1* mutant compared to other genotypes. Taken together, these data indicate that AvrRps4N-mediated delivery of PopP2 from *Pseudomonas* can trigger RPS4/RRS1-dependent HR and immunity in Arabidopsis.

We further tested if *Pseudomonas*-delivered PopP2 recognition requires a specific subcellular localization, as reported for AvrRps4 [8]. We engineered a PopP2^149–488^ variant lacking the native NLS, to carry a NLS or a nuclear export signal (NES) tag at the C-terminus. The avirulence activity of these PopP2 variants was tested in two resistant transgenic Arabidopsis lines, RLD (RPS4^Ler^) and Col-0 (RRS1^Ws-2^). Pf0-1(T3S)-delivered PopP2^NES^, failed to trigger HR in both transgenic lines and in wild type Ws-2, despite being expressed during plant infection, indicating that nuclear localization of PopP2 is required to trigger RPS4/RRS1-dependent HR (Figure S2A, S2E and S3). The PopP2^NES^ variant induced a response comparable to PopP2^C321A^, an enzymatically inactive variant that does not trigger RPS4/RRS1-R-dependent immunity [36] in wild type Ws-2 when HR-inducing activity was quantified by ion leakage measurements (Figure S2B). We could also show that PopP2^NES^, in contrast to PopP2^NLS^, could not restrict the virulence of bacteria when delivered from *Pto* DC3000, nor trigger expression of defense genes when delivered from Pf0-1(T3S) (Figures S2C and S2D). As these data suggest that PopP2 triggers HR and immunity in the nucleus, we independently assessed previously reported AvrRps4 variants [8]. Unexpectedly, both AvrRps4^NLS^ and AvrRps4^NES^ variants triggered HR and elevated ion leakage in the Ws-2 accession when delivered from Pf0-1(T3S) (Figure S2B and S2E).

### *Pseudomonas*-delivered PopP2 induces RRS1-R- and acetyltransferase activity-dependent transcriptional changes early after bacterial infiltration

RRS1 is a TIR-NB-LRR protein with a WRKY DNA binding domain, which belongs to Group III of the WRKY superfamily [37]. RRS1^SLH1^, which carries a leucine insertion near the WRKY motif, shows strongly reduced DNA binding by its WRKY domain [30]. This reduced DNA binding correlates with auto-immunity of the *slh1* mutant, suggesting a critical role of RRS1 in transcriptional regulation of defense genes. Delivery of PopP2 from *Pseudomonas via* T3S, combined with the RPS4/RRS1-R dependence of this PopP2-triggered HR, enables direct assessment of RRS1-R-dependent transcriptional regulation. To identify PopP2-triggered and RPS4/RRS1-dependent early transcriptional responses, genome-wide expression profiling was carried out using EXPRSS, an Illumina sequencing based method [38]. Wild type Ws-2 and *rrs1-1* plants were infiltrated with Pf0-1(T3S) delivering PopP2^WT^ or PopP2^C321A^. The infiltrated leaf tissues were collected at 2, 4, 6 and 8 hpi for total RNA extraction, as onset of HR began at 8 hours after bacterial infiltration in an incompatible interaction (PopP2^WT^/Ws-2).

**Figure 2:**
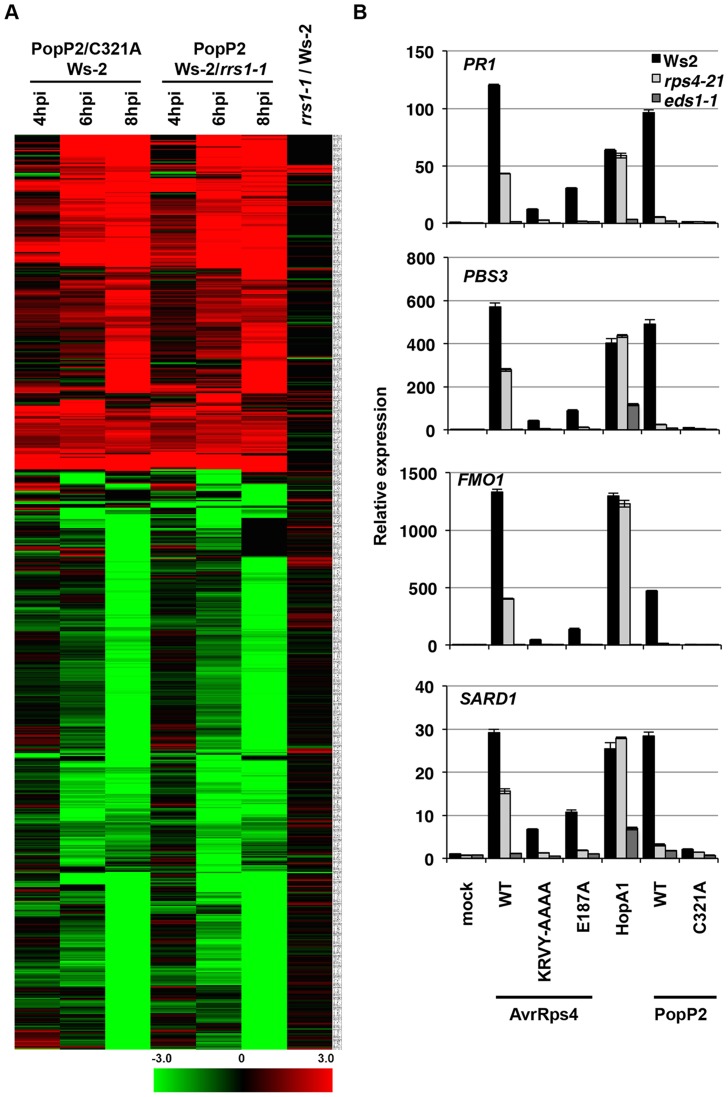
*Pseudomonas*-delivered PopP2 induces RRS1- and acetyltransferase activity-dependent transcriptional changes early after bacterial infection. (*A*) Hierarchical clustering of RRS1- or PopP2-dependent gene expression. Fold-change values of 719 genes (differentially expressed at least in one time point) from all time points show the predominance of gene induction at early time points. Black, red and green colours indicate no change, up-regulated and down-regulated, respectively. C321A, an inactive PopP2 variant carrying an Alanine mutation at one of the catalytic core residues, Cysteine 321 (*B*) Confirmation of selected PopP2-induced genes by qRT-PCR. Five week-old plants were infiltrated with Pf0-1(T3S) expressing the indicated AvrRps4, HopA1 or PopP2 variants. Samples were taken at 8 hpi for total RNA extraction. The numbers on the Y-axis indicate fold induction compared to mock treated samples.

For differential expression analysis, PopP2^WT^-infiltrated Ws-2 samples were compared either to PopP2^C321A^ mutant on Ws-2 or PopP2^WT^ on *rrs1-1*. Essentially complete overlap was observed between the differentially regulated genes in the two comparisons (Figure 2A), consistent with our results showing that Pf0-1(T3S)-delivered PopP2 triggers RRS1- and acetyltransferase activity-dependent immunity (Figures 1 and S2). In total, 719 genes were differentially expressed in an RRS1- and acetyltransferase activity-dependent manner in at least one of the time points surveyed (Table S1). Gene ontology enrichment analysis using ATCOECIS [39] showed that most of the up-regulated genes are involved in defense, while most of the down-regulated genes are involved in photosynthesis and enriched in chloroplast-localized genes (Table S2). Interestingly, the majority of genes differentially expressed at 4 and 6 hpi were up-regulated, while many down-regulated genes were observed at 8 hpi (Figure 2A). The early (4 and 6 hpi) up-regulated genes, such as *SID2*, *FMO1*, *NudT7*, *PBS3* and *PAD4*, have previously been implicated in plant defense (Table S3). Further analysis of mean expression of genes induced at 4 and 6 hpi (Table S3) showed that there was greater gene induction in Ws-2 infiltrated with PopP2^WT^ (∼20–100 fold) than in Ws-2 infiltrated with PopP2^C321A^ or in *rrs1-1* infiltrated with PopP2^WT^ (∼2–10 fold). For simplicity, we interpret genes induced by PopP2^C321A^ as induced by the repertoire of PAMPs in Pf0 (thus, PTI-induced), and by PopP2^WT^ as PTI+ETI-induced. To validate our transcriptional expression profiling results, we performed quantitative RT-PCR (qRT-PCR) verification of *EDS5*, *NudT6*, *WRKY18* and *WRKY40* with the cDNA used for Illumina libraries. Expression of *EDS5* and *NudT6* but not *WRKY18* and *WRKY40* was specifically regulated by ETI in our expression profiling data. In qRT-PCR experiments, PopP2 but not PopP2^C321A^ variant delivered from Pf0-1(T3S) induced *EDS5* and *NudT6* in an RRS1-dependent manner, while expression of *WRKY18* and *WRKY40* was induced in the absence of ETI (Figure S4).

AvrRps4- and PopP2-dependent transcriptional changes in resistant plants have been investigated previously [28], [29], [40]. We compared these available micro-array and RNA-seq data with our results. To minimize the effects of experimental and technical differences from the AvrRps4/Ws-2 data [28], genes altered in expression at 6 hpi due to mock treatment were subtracted from the comparison; similarly, only the GMI1000/GMI1000ΔPopP2-infected Nd-1 data were used from the Hu *et al*. [40] study. For comparative analysis the differential expression from PTI, PTI+ETI and ETI responses were combined for data presented in this study (Table S1) and the data from Howard *et al.* [29]. A summary of these comparisons is presented in Figure S5 and details of genes from comparative datasets are presented in Table S4. Transcriptional changes upon AvrRps4 infection on Col-0 and Ws-2 [28], [29] considerably overlapped with PopP2-regulated genes identified both in our study and the GMI1000/GMI1000ΔPopP2 study [40] (Figure S5). The majority of early PTI+ETI-induced genes detected in our study were also found to be AvrRps4-responsive [28], [29] (Figure S5 and Table S4).

We next tested the expression of four PopP2-responsive genes *PBS3*, *SARD1*, *FMO1* and *PR1* by qRT-PCR in Ws-2, *rps4-21* and *eds1-1*. At 8 hpi, Pf0-1(T3S)-delivered AvrRps4^WT^, HopA1 or PopP2^WT^ triggered similar levels of induction of the four genes in Ws-2 (Figure 2B). Induction of all four genes was strictly dependent on EDS1 and abolished when non-functional variants of the effectors (AvrRps4^KRVY-AAAA^, AvrRps4^E187A^ and PopP2^C321A^) were delivered. PTI+ETI-induction of all four genes in response to PopP2 was reduced to PTI-induced levels in both *rps4-21* and in *rrs1-1* mutants, confirming RPS4/RRS1-R-dependence of PopP2-induced transcriptional changes. AvrRps4-triggered induction of all four genes was reduced but not abolished in the *rps4-21* mutant, likely due to RPS4-independent recognition of AvrRps4 in Ws-2 [26], [41]. These expression profiling data thus reveal the genes specifically regulated at very early stages of PopP2-triggered, RPS4/RRS1-dependent immunity in Arabidopsis. Moreover, these ETI transcriptional changes are very similar after AvrRps4 or PopP2 recognition.

### Expression profiles of RPS4/RRS1-dependent responses to AvrRps4 or PopP2, and of Arabidopsis RRS1^SLH1^ mutant temperature shift, substantially overlap

**Figure 3:**
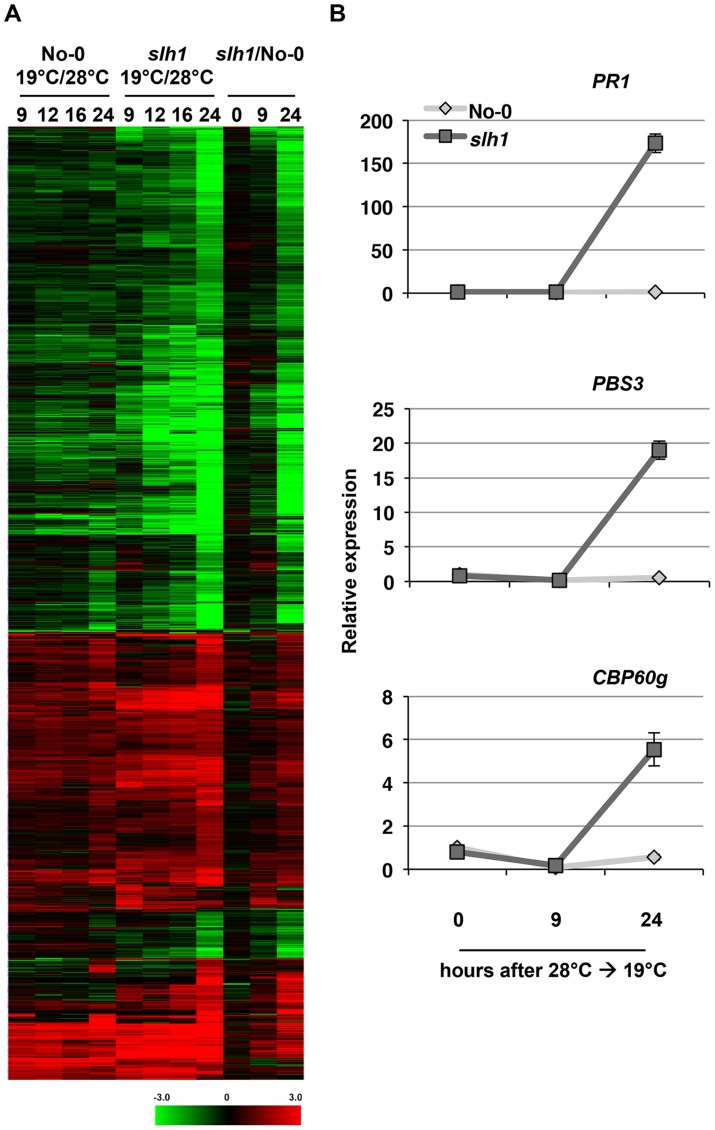
Low temperature-dependent transcription profiling of the *slh1* mutant. (*A*) Hierarchical clustering of No-0 and *slh1* temperature-dependent differential gene expression. Fold-change values of 5611 genes (differentially expressed at least in one time point) are shown. The numbers on top of the heat map indicate the time (h) after temperature shift. Black, red and green colours indicate no change, up-regulated and down-regulated, respectively. (*B*) qRT-PCR analysis of selected RRS1^SLH1^-regulated genes following the temperature shift (28°C to 19°C) in 4 week-old No-0 and *slh1* plants. Transcript accumulation is presented relative to No-0 before temperature shift (28°C).

To compare *slh1* aberrant defense responses to effector-triggered RPS4/RRS1-mediated immunity, we conducted transcription profiling of the *slh1* mutant over a time course after shifting plants from 28°C to 19°C, using Illumina tag sequencing [38]. A total of 1821 genes showed temperature-dependent differential expression in RRS1^SLH1^ after 24 hours (h) compared to wild type No-0 (Figure 3A). We confirmed the temperature-dependent regulation of 3 genes with differential induction in *slh1* by qRT-PCR. *PR1*, *PBS3* and *CBP60g* transcript accumulation was induced in *slh1* plants between 9 and 24 h after the shift from 28°C to 19°C whereas it was unaltered in temperature-shifted No-0 plants (Figure 3B).

**Figure 4:**
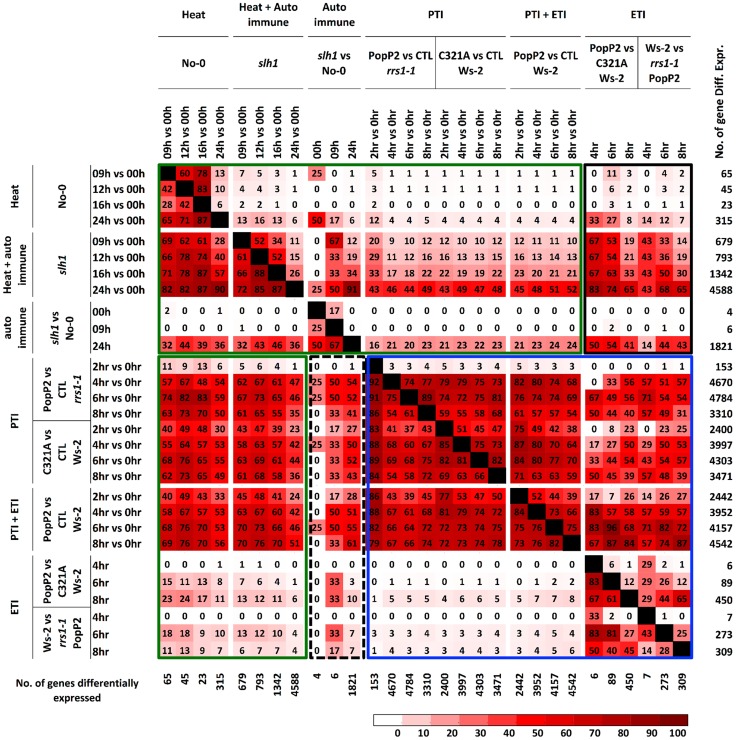
Percentage pairwise overlap of genes differentially expressed during the time course of PopP2 or PopP2^C321A^ on Ws-2 and *rrs1-1* and the time course of temperature shift on No-0 and *slh1*. Each time course response is categorized based on underlying response (PTI, ETI, temperature shift, auto-immunity and combinations). Each cell represents percentage of genes differentially expressed from the column experiment that were also differentially expressed in the row experiment. Green boxes highlight genes regulated by heat stress and PTI, PTI+ETI responses; blue box highlights genes regulated by PTI, ETI and PTI+ETI; black boxes highlight genes regulated by auto-immunity, heat stress and by PTI, ETI and PTI+ETI. The number of gene differentially expressed in each time course is indicated on the right. PTI, PopP2^C321A^-regulated genes; ETI, PopP2^WT^- but not PopP2^C321A^-regulated genes; temperature shift, temperature shift-regulated genes in No-0 wild-type; auto-immunity, temperature shirt-regulated genes in *slh1* mutant but not in No-0 wild-type.

We compared the *slh1*/No-0 temperature-shift transcriptional dataset to the PopP2/RRS1-time course dataset by analyzing the pairwise overlap of genes differentially expressed in both experiments (Figure 4). Each time course response was categorized according to the mode of elicitation as PTI, ETI, temperature shift, auto-immunity, or corresponding combinations (e.g. PTI+ETI). We found that most (∼83%) of the PopP2/RRS1 ETI genes were differentially expressed in *slh1* auto-immune and temperature shift responses, while up to 54% of ETI genes were also differentially expressed in the auto-immune response but not by temperature shift (Figure 4, black box). Similarly, more than 55% of auto-immune genes were also differentially expressed in PTI and PTI+ETI (Figure 4, dotted block box). Most ETI genes were also differentially expressed in PTI+ETI (more than 85%) and in PTI (up to 70%). However, less than 10% of the PTI genes were differentially expressed during ETI (Figure 4, blue box). This strongly suggests that many ETI responses involve potentiation of a subset of PTI responses, with few genes solely regulated by effector recognition. The ETI-specific genes that are regulated in PopP2 acetyltransferase activity- and RRS1-dependent manner include nucleotide/ATP-binding protein encoding genes such as NB-LRRs (Table S1).

Similarly, we found that most temperature shift-regulated genes (up to 83%) (Table S5) were also differentially expressed by PTI or PTI+ETI, but only 25% were specifically affected by ETI, and less than 5% of the PTI-responsive genes were differentially expressed by temperature shift (Figure 4, green box). Up to 50% of PTI or PTI+ETI genes were also differentially expressed by temperature shift and auto-immune response, while about 25% of PTI or PTI+ETI genes were differentially expressed by auto-immune response (Figure 4, green box). These results indicate that PTI broadly activates genes responsive to heat, auto immunity and ETI.

These analyses indicate that *slh1* auto-immunity overlaps strongly with PopP2- and RPS4/RRS1-R-dependent ETI. Thus, RRS1^SLH1^-induced transcriptional reprogramming results in similar gene expression changes to those observed in AvrRps4- or PopP2-triggered immunity, indicating that the *slh1* lethal phenotype mimics RPS4/RRS1-dependent ETI at the transcriptional level.

### Identification of *sushi* (*suppressor of* slh1 *immunity*) mutants

**Figure 5:**
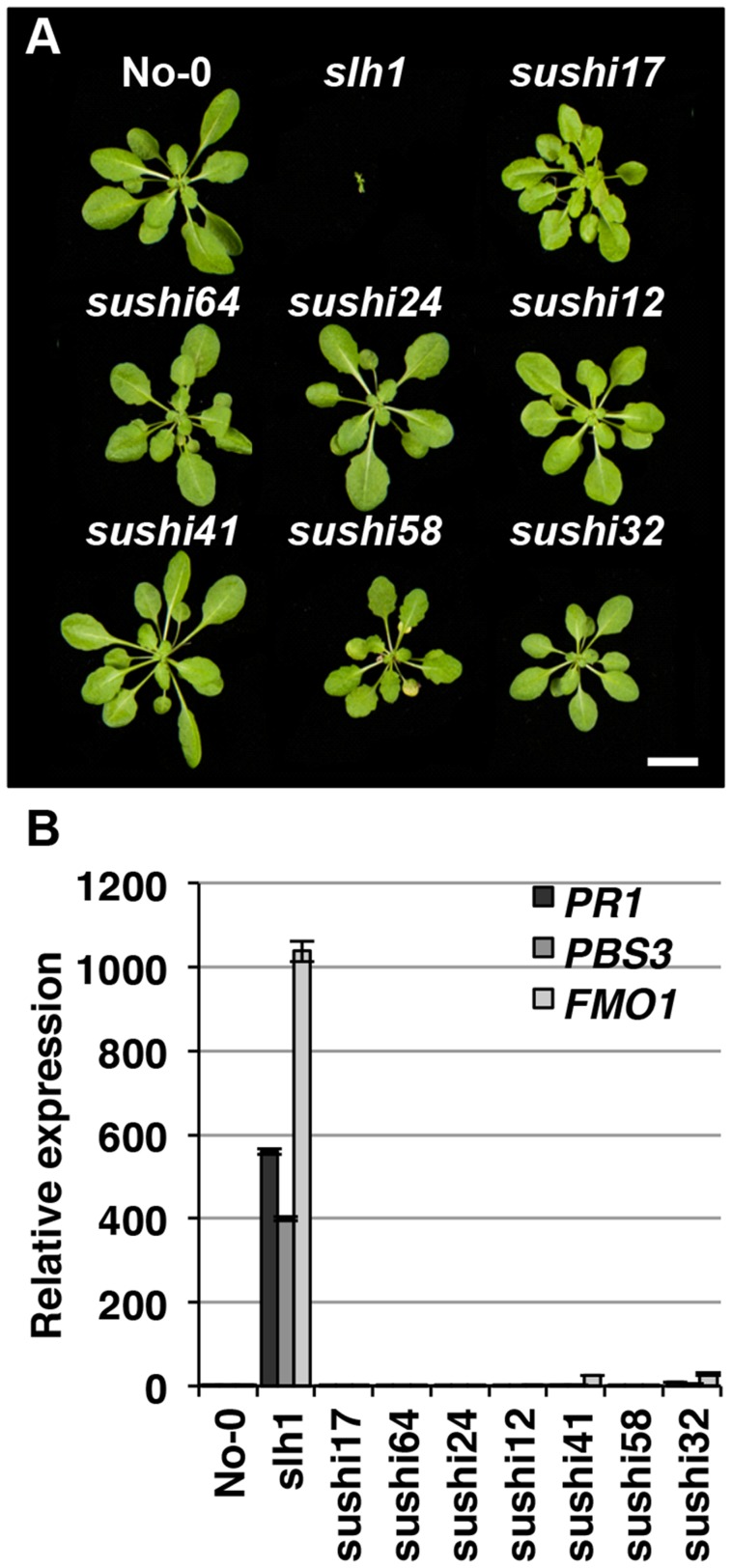
Identification of *sushi* (suppressor of *slh1* immunity) mutants. Fully rescued *sushi* mutant (M3), wild type No-0 and *slh1* plants were grown at 21°C under short-day condition for four weeks. (*A*) Plant morphology. (*B*) qRT-PCR analysis of selected RRS1^SLH1^-regulated genes. Transcript accumulation is presented relative to No-0.

Lethality of *slh1* at 21°C is correlated with constitutive activation of defense responses including high expression of *Pathogenesis Related* (*PR*) genes and SA accumulation [30]. We hypothesized that mutations that affect RRS1^SLH1^-mediated signaling components or RRS1^SLH1^ expression would suppress *slh1* lethality. To identify genetic components required for RRS1^SLH1^-dependent immunity, we conducted a suppressor screen. *slh1* seeds were incubated with ethyl methanesulfonate (EMS), ∼7,000 M1 plants were grown at 28°C and M2 seeds were harvested. By screening ∼500,000 M2 mutant plants at 21°C, we identified 83 families with a *suppressor of* slh1 *immunity* (*sushi*) mutant phenotype. Among them, 69 and 14 could rescue the *slh1* lethal phenotype to a wild type-like and an improved morphology, respectively. We further analyzed the progeny of 7 selected fully rescued *sushi* mutants for morphological development and defense marker gene expression in the M3 generation (Figure 5). Growth of *sushi* mutants at 21°C was similar to wild type No-0, whereas *slh1* plants did not develop beyond the first true leaf stage (Figure 5A). *PR1*, *PBS3* and *FMO1* expression was elevated in *slh1* mutants grown constantly at 21°C or 24 h after shift from 28°C to 21°C, but not in fully rescued *sushi* mutants (Figures 5B and S6).

To exclude any contamination with wild type seeds, we confirmed the presence of the *slh1* mutation in 72 of the 83 M3 individual *sushi* mutants identified using a cleaved amplified polymorphic sequences (CAPS) marker [30]. Next, we carried out Sanger sequencing of *RRS1* and *RPS4* coding regions in these mutants. As expected from the complete suppression of the *slh1* phenotype, we identified 6 *sushi* intragenic suppressor mutants that carry an early stop codon in *RRS1^SLH1^* and 8 other non-synonymous mutations (Table S6). Surprisingly, non-synonymous mutations were also identified throughout the *RPS4* coding region in 34 rescued *sushi* mutants (Table S6). Most of the altered amino acid residues have not previously been shown to be required for *RPS4* function [24]. However, *sushi52* and *sushi22* harbour non-synonymous mutations at positions R28 and E88 that are important for RPS4^TIR+80^-triggered HR in tobacco [42], further verifying the crucial role of the RPS4 TIR domain function in RRS1^SLH1^-mediated defense activation.

It was previously reported that mutations in SID2/ICS1/EDS16 or SID1/EDS5 result in suppression of the *RRS1^SLH1^* mutant phenotype [30]. We sequenced the coding region of these genes in the non-*RRS1*, non-*RPS4* mutants, and found one *sushi* mutant that carried a mutation in SID2/ICS1/EDS16 (*sushi70*, Table S6), and no mutants that carry mutations in SID1/EDS5. Similarly to Arabidopsis accession Col-0, wild type No-0 carries two copies of *EDS1*. Therefore, EDS1 coding sequence was not verified in the *sushi* lines. The 23 remaining unassigned *SUSHI* mutations are now subjected to further analysis to identify new signaling components of RRS1^SLH1^-mediated immunity.

### RPS4 is required for activation of RRS1^SLH1^-mediated immunity

**Figure 6:**
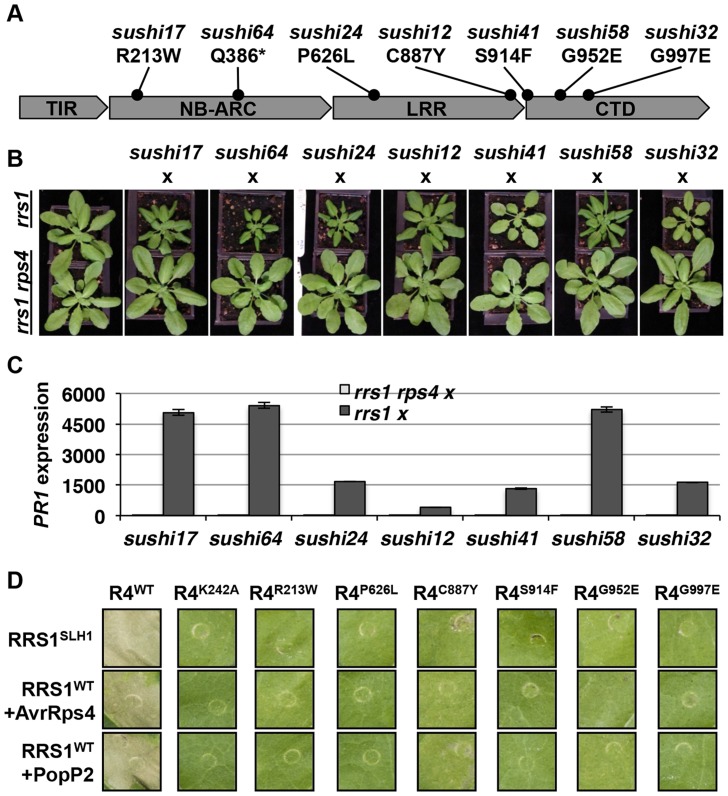
RPS4 is required for RRS1^SLH1^-mediated activation of immunity. (*A*) Schematic presentation of *SUSHI* mutations in RPS4. The asterisk indicates premature stop codon. (*B*) The RRS1^SLH1^-induced growth restriction phenotype of *sushi* mutants is RPS4-dependent. The F1 hybrids between *rrs1-1* or *rrs1-1 rps4-21* and *sushi* were grown for five weeks at 21°C before the photograph was taken. (*C*) Growth restriction of F1 hybrids (shown in (*B*)) correlates with *PR1* transcript accumulation as determined by qRT-PCR. *PR1* transcript accumulation is presented relative to *rrs1-1* and *rrs1-1 rps4-21* respectively. (*D*) RPS4^SUSHI^ variants do not confer RRS1^SLH1^-induced hypersensitive response or recognition of AvrRps4 or PopP2 when transiently expressed in tobacco leaf cells. Photographs were taken 3 days after agroinfiltration.

Homo- or hemizygous, but not heterozygous, No-0 plants carrying *RRS1^SLH1^* display a stunted phenotype at 21°C due to elevated immunity [30]. To verify that *RPS4* is required for RRS1^SLH1^ function, we crossed 7 *sushi* lines carrying mutations in *RPS4* (*sushi17*, *64*, *24*, *12*, *41*, *58* and *32*) to *rrs1-1* and *rrs1-1 rps4-21* knockout mutants [26]. The resulting F1 individuals from both crosses were hemizygous *RRS1^SLH1^/rrs1* for *RRS1* locus (Figure S7) and either *RPS4^sushi^/RPS4^WT^* or *RPS4^sushi^/rps4* at the *RPS4* locus. As expected, the F1 plants derived from a cross between the *sushi* and *rrs1-1* were stunted and showed elevated *PR1* expression level (Figure 6A–C). These phenotypes were both completely suppressed in the F1 plants derived from a cross between *sushi* mutants in *RPS4* and *rrs1-1 rps4-21* double mutant. This result confirms that *RPS4* is required for *RRS1^SLH1^*-mediated activation of immunity.

To further verify the functional requirement for *RPS4* in *RRS1^SLH1^*-mediated immunity, we recapitulated RRS1^SLH1^-mediated defense activation in *Nicotiana tabacum*. As shown recently [39], *Agrobacterium*-mediated transient co-transformation (hereafter, agroinfiltration) of RPS4-HA, RRS1-His-Flag and wild type AvrRps4-GFP or PopP2-GFP induced strong HR within 3 dpi (Figure S8A). The specificity of recognition was further verified by comparing functionally characterized mutant variants of AvrRps4 or PopP2 to wild type. As expected, AvrRps4^E187A^, AvrRps4^KRVY-AAAA^ and PopP2^C321A^ variants did not induce RPS4/RRS1-dependent HR in tobacco (Figure S8A). We have also verified that AvrRps4 and PopP2 recognition in tobacco activate defense genes orthologous to those that are regulated by RRS1 in Arabidopsis. The transcripts of the defense genes *NtWRKY51* and *NtNudT7* were highly up regulated when PopP2-GFP was co-expressed with RPS4-HA and RRS1-His-Flag in tobacco (Figure S8B). Agroinfiltration of GFP or PopP2^C321A^-GFP with RPS4-HA and RRS1-His-Flag induced significantly lower accumulation of defense gene transcripts compared to wild type PopP2 (Figure S8B). Taken together, these results further demonstrate that our transient agroinfiltration assay can also be used to investigate RPS4/RRS1 regulated immunity.

Agroinfiltration of epitope-tagged RRS1^SLH1^-His-Flag and RPS4^WT^-HA triggered HR in tobacco leaf cells, whereas RRS1^SLH1^ co-expressed with GFP or RPS4^K242A^ (P-loop mutant) did not (Figures 6D and 8B). Consistent with our Arabidopsis genetic data (Figure 6B), agroinfiltration of RRS1^SLH1^ with each RPS4^SUSHI^ variant did not trigger HR in tobacco (Figure 6D). Protein accumulation of the 7 tested RPS4^SUSHI^ variants was comparable to that of RPS4^WT^, indicating that the lack of HR was not due to low protein expression levels (Figure S9). Moreover, as expected from our genetic analysis, RPS4^SUSHI^ variants did not have a dominant negative effect on RPS4^WT^ function, when both were co-expressed with RRS1^SLH1^ (Figure S10). We then tested whether *SUSHI* mutant alleles of *RPS4* confer RRS1-dependent recognition of AvrRps4 or PopP2. Agroinfiltration of RRS1^WT^, RPS4^WT^ and either AvrRps4 or PopP2, triggered RPS4 P-loop-dependent HR in infiltrated tobacco leaf sectors [43] (Figure 6D). Importantly, agroinfiltration of the 7 RPS4^SUSHI^ variants did not confer responsiveness to AvrRps4 or PopP2 (Figure 6D). Taken together, these data show that *RPS4* is required for *RRS1^SLH1^*-mediated and Avr-triggered/*RRS1*-dependent defense signaling activation. Recently, we showed the physical interaction of RRS1 and RPS4 [43]. We hypothesized that RPS4^SUSHI^ variants may have lost their ability to interact with RRS1^SLH1^. However, RPS4^SUSHI^-HA variants and RPS4^WT^-HA were co-immunoprecipitated by RRS1^SLH1^-Flag or RRS1^WT^-Flag (Figure S9A-B). This result suggests that RPS4-RRS1 interaction is insufficient for signaling activation.

**Figure 7:**
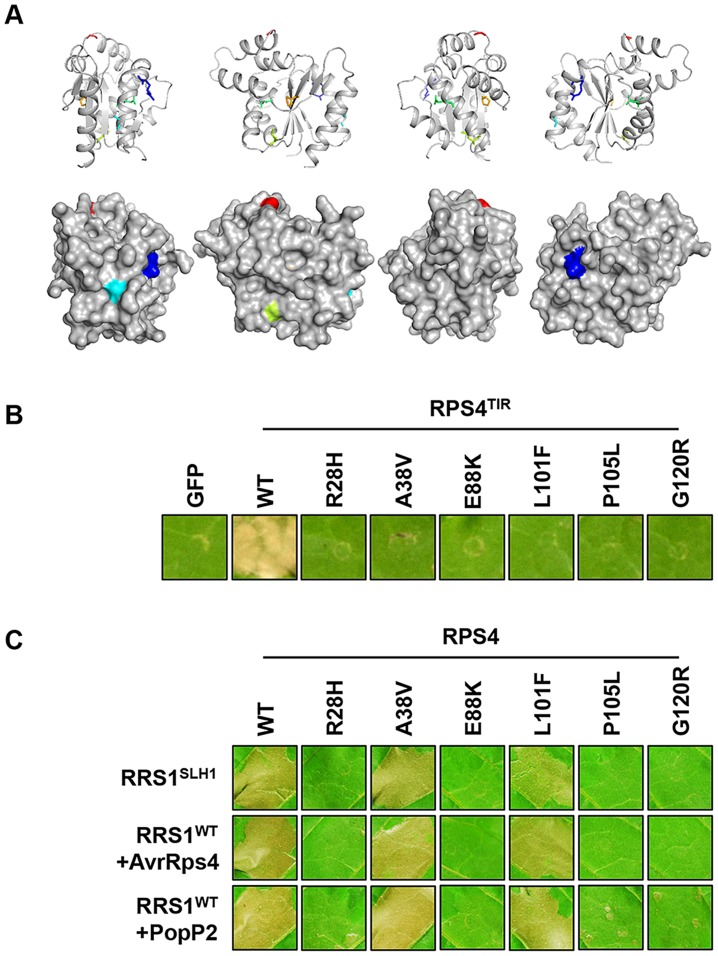
Functional analysis of *SUSHI* mutations in the RPS4 TIR domain. (*A*) *SUSHI* mutations within the RPS4 TIR domain structure (PDB ID 4c6r) in cartoon (top) and surface (bottom) representation (figures were generated using PyMOL (Delano Scientific)). Molecules are rotated 90° around the y-axis from left to right. Mutated residues are labelled R28 (Blue – *sushi52*), A38 (Teal – *sushi14*), E88 (Green – *sushi22*), L101 (Lime – *sushi71*), P105 (Orange – *sushi89*) and G120 (Red – *sushi29*). (*B*) The *SUSHI* mutations abolish RPS4 TIR-induced HR in tobacco agroinfiltration assay. (*C*) Analysis of the full-length RPS4 variants carrying *SUSHI* mutations in the TIR domain for recognition of AvrRps4 or PopP2 in tobacco agroinfiltration assay. The photographs were taken 3 days after agroinfiltration.

We identified six additional *sushi* mutants that carry mutations in the TIR domain of RPS4, the structure of which is known [43]. The stunted growth and elevated defense transcript accumulation of *slh1* at 21°C were considerably suppressed in *sushi52* (R28H), *14* (A38V), *22* (E88K), *71* (L101F), *89* (P105L) and *29* (G120R) (Figure S11). The RPS4 TIR domain structure suggests that side-chains from R28 and A38 are surface exposed, while the side-chains of the other mutated residues are buried (Figure 7A). RPS4^TIR^ expression is sufficient to trigger HR in tobacco after agroinfiltration (Figure 7B) [42]. Therefore we introduced these six *SUSHI* mutations into an RPS4^TIR^ construct (amino acids 1 to 250) to test their individual effect on RPS4 TIR domain signaling. Strikingly, all six mutations suppressed this response, suggesting that each of these residues is important for RPS4 TIR domain defense activation either through interaction with downstream partners or by maintaining the correct signalling-competent structural conformation, as the protein stability/accumulation was not significantly altered when expressed as GFP fusions in tobacco (Figure S12D). Intriguingly, when *SUSHI* mutations were tested in the RPS4 full-length context by co-expression in tobacco with RRS1 and the effectors, A38V and L101F did not suppress RRS1^SLH1^- nor AvrRps4- and PopP2-triggered HR (Figure 7C). This discrepancy was not due to inconsistent level of protein accumulation (Figure S12E) but might illustrate a limitation of the transient expression system in tobacco, or subtle differences between defense activation by RPS4^TIR^, and by the activated RPS4/RRS1 complex.

### RPS4 and RRS1 properties required for RRS1^SLH1^-mediated hypersensitive response in tobacco

**Figure 8:**
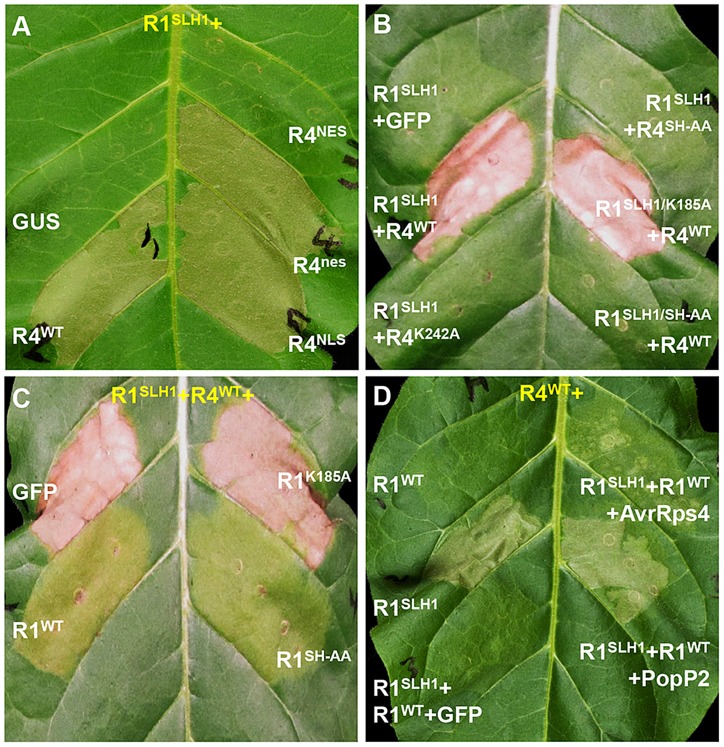
Characterization of RRS1^SLH1^-induced hypersensitive response. RRS1 (R1), RPS4 (R4), AvrRps4 (A4) and PopP2 (P2) were C-terminally epitope-tagged with His-Flag, HA, GFP and GFP, respectively. The photographs were taken 3 days after agroinfiltration. (*A*) The nuclear localization of RPS4 is required for RRS1^SLH1^-induced HR. NES and NLS indicate nuclear export signal and nuclear localization signal, respectively. (*B*) RRS1^SLH1^-dependent HR requires RPS4 P-loop (K242A) and TIR-TIR domain heterodimerization (SH-AA) but not RRS1 P-loop (K185A). (*C*) The interference of RRS1^SLH1^-induced HR by wild type RRS1 requires the P-loop but not the SH-motif. (*D*) RRS1^SLH1^ does not fully interfere with AvrRps4 or PopP2 recognition by wild type RRS1.

As nuclear localization of RPS4 is necessary for AvrRps4-triggered immunity [41], we investigated the role of RPS4 nuclear localization in RRS1^SLH1^-mediated cell death. Co-expression of RRS1^SLH1^ with RPS4^WT^ or RPS4^NLS^ induced HR (Figure 8A). However, RPS4^NES^ did not induce HR when co-expressed with RRS1^SLH1^, indicating the importance of RPS4 nuclear localization for RRS1^SLH1^ function, consistent with a previous report [41]. Nucleotide binding to the invariant Lys residue of the P-loop motif in the NB domain of R proteins is critical for conformational change and immunity activation [4], [44], [45]. Agroinfiltration of RPS4^WT^, but not the P-loop mutant RPS4^K242A^, triggered HR when co-expressed with RRS1^SLH1^ (Figure 8B). However, RPS4^K242A^ does interact with RRS1^SLH1^ and RRS1^WT^ (Figure S9C). Therefore, a functional RPS4 P-loop motif is required for activation of RRS1^SLH1^-induced defense but is not an absolute requirement for RPS4-RRS1 interaction. Surprisingly, introduction of the P-loop mutation (K185A) in the RRS1^SLH1^ protein sequence did not affect HR-inducing activity when co-expressed with RPS4^WT^ (Figure 8B). Thus, P-loop motif-dependent conformational change may not be required for defense activation by RRS1^SLH1^, consistent with the functionality of an RRS1 P-loop mutant in AvrRps4 or PopP2 recognition [43].

Structural analysis of RPS4 and RRS1 TIR domains revealed an “SH motif” in regions that mediate heterodimerization between RPS4 (S33 and H34) and RRS1 (S25 and H26) [43]. Moreover, RPS4 or RRS1 variants carrying a mutated SH motif (SH-AA) cannot recognize AvrRps4 or PopP2 in tobacco agroinfiltration [43]. To investigate if TIR-TIR domain heterodimerization is also required for RRS1^SLH1^ function, SH-AA mutations were introduced in RPS4^WT^ and RRS1^SLH1^ variants. Agroinfiltration of RRS1^SLH1^ and RPS4^SH-AA^, or RRS1^SLH1/SH-AA^ and RPS4^WT^ did not induce HR in tobacco suggesting that TIR-TIR domain heterodimerization between RRS1 and RPS4 is required for RRS1^SLH1^-dependent defense activation (Figure 8B). However, in the context of the full-length proteins the RRS1^SLH1/SH-AA^ variant could still interact with RPS4^WT^ (Figure S9C).

### Co-expression of RRS1 with RPS4/RRS1^SLH1^ suppresses HR, consistent with the recessive nature of RRS1^SLH1^

RRS1^SLH1^-dependent lethality is recessive [30]. In agreement, agroinfiltration of RRS1^WT^ but not of GFP interfered with HR induced by co-expression of RRS1^SLH1^ and RPS4^WT^ in tobacco (Figure 8C–D). Interestingly, the RRS1^K185A^ variant did not interfere with RRS1^SLH1^-induced HR whereas the RRS1^SH-AA^ variant did (Figure 8C), indicating that nucleotide-binding function but not RPS4/RRS1 TIR-TIR domain interaction is required for RRS1-mediated interference with RRS1^SLH1^-induced HR. These agroinfiltration data are consistent with our transcriptomic and genetic analyses and demonstrate the striking similarity of RRS1^SLH1^ and Avr-triggered/RRS1-dependent defense activation.

As RRS1^SLH1^/RPS4-dependent constitutive HR is prevented by co-expression of RRS1^WT^, we tested if RRS1^SLH1^ interferes with RRS1^WT^ recognition of AvrRps4 or PopP2. Interestingly, in the presence of both RRS1 variants and RPS4, AvrRps4- or PopP2-triggered HR is still observed suggesting that RRS1^SLH1^ did not completely abolish RRS1^WT^ function (Figure 8D). However, AvrRps4-triggered HR was attenuated considerably compared to PopP2-triggered HR under the same conditions (Figure 8D).

## Discussion

Although both AvrRps4 and PopP2 are recognized by RPS4 and RRS1, a thorough comparison of immune responses, particularly of early transcriptional changes, has been difficult due to the distinct infection modes of the bacterial pathogens from which AvrRps4 (*Pseudomonas syringae*) and PopP2 (*Ralstonia solanacearum*) originate. Root infection of Arabidopsis plants with *R. solanacearum* causes wilting within 2 weeks, whereas *Pseudomonas*-delivered AvrRps4 triggers HR in Arabidopsis Ws-2 leaf cells within 24 hours. PopP2 delivery from Pf0-1(T3S) allowed us to compare the transcriptional reprogramming caused by recognition of AvrRps4 or PopP2 at the earliest stages and has resulted in the identification of a set of similarly regulated ETI-specific genes. It is interesting that the NLS is dispensable for the avirulence activity of PopP2 in our assays. It was shown that removal of the N-terminal NLS renders localization of PopP2 and co-expressed RRS1-S/R variants nuclear-cytoplasmic [46]. However, the significance of this PopP2 NLS-dependent relocalization of RRS1 is not known, as there have been no studies showing ETI phenotypes triggered by PopP2 lacking the NLS. As shown in Figure S2, a PopP2 variant lacking an N-terminal NLS shows similar levels of avirulence compared to wild type. Thus, PopP2 NLS-dependent relocalization of RRS1 may not be significant in PopP2-triggered immunity. Alternatively, the portion of RRS1 that is localized in the nucleus with the NLS lacking PopP2 might be sufficient to activate ETI.

It is intriguing to find that AvrRps4^NES^ and AvrRps4^NLS^ are comparable in their ability to elicit HR in Arabidopsis Ws-2 (Figure S2E). AvrRps4^NES^ triggers a slightly lower ion leakage level than AvrRps4^NLS^ (Figure S2C). We conclude that regardless of AvrRps4 contribution to defense activation in the cytoplasm, its major role is in the nucleus *via* interactions with the RPS4/RRS1 complex.

*Pseudomonas* T3S delivery of PopP2 provides a useful tool to investigate RPS4/RRS1-dependent transcriptional regulation at an early stage of ETI. In addition, by comparing non-functional variants of AvrRps4 and PopP2 to wild type proteins, we could identify the genes whose transcriptional changes were specific to Avr function. As Pf0-1(T3S) carries a mutated *HopA1* gene which is unable to trigger RPS6-dependent immunity in Arabidopsis, the gene expression change in *rrs1-1* infiltrated with PopP2^WT^ or in Ws-2 infiltrated with PopP2^C321A^ can be considered as PTI resulting from perception of the Pf0-1 PAMP repertoire. We thus report defense gene expression changes as PTI vs. PTI+ETI (Table S3). Gene ontology enrichment has shown that the majority of early up-regulated genes are involved in plant defense.

Comparative analysis with previously published microarray data shows that many PopP2-triggered early gene expression changes overlap substantially with AvrRps4-triggered transcriptional regulation [28], [29]. It is interesting to note that PopP2-regulated genes also overlap substantially with previously reported PopP2-induced genes at a later stage of infection when delivered from *R. solanacearum* [40]. Our discovery of early responding genes will allow us to test if they are directly regulated by RPS4/RRS1. It has been recently shown that WRKY18 and WRKY40 positively contribute towards AvrRps4-triggered immunity [47]. Consistent with this, WRKY18 and WRKY40 were highly induced at 3 and 6 hpi by AvrRps4 (Table S4). However, our experimental design enabled us to show that both WRKY18 and WRKY40 are primarily induced due to PTI (Figure S4). PTI+ETI and PTI induction of WRKY40 expression are indistinguishable. There is slightly higher PTI+ETI-induced expression of WRKY18 in response to PopP2^WT^ in Ws-2 at later time points (6 and 8 hpi) compared to PTI elicited by PopP2^C321A^ in Ws-2 or PopP2^WT^ in *rrs1-1* (Figure S4), but this could be due to elevated SA levels that we presume are responsible for strong *PR1* induction at 8 hpi.

It is interesting to note that AvrRps4-induced regulation of ETI genes only partially requires RPS4. This is consistent with AvrRps4 recognition being conferred by both RPS4/RRS1-dependent and -independent mechanisms. Identification of an *R* gene(s) that confer RPS4/RRS1-independent immunity will enable comparative analysis of how AvrRps4-induced ETI genes are transcriptionally regulated by different *R* genes. It was remarkable to observe that AvrRps4, PopP2 and HopA1 induced common genes at early stage of defense activation, suggesting a possible EDS1-dependent conserved gene activation mechanism in ETI.

Several auto-active alleles of NB-LRR genes have been found [9], [10], [30], [48], [49], though unlike the recessive *slh1*, all others are dominant or semi-dominant. Plants carrying an auto-active *R* gene typically show temperature-dependent lethality and enhanced resistance to virulent pathogens [30]–[32]. However, in many cases the overlap between elevated disease resistance that is conferred by an auto-active *R* gene allele and by Avr-triggered immunity is poorly defined. Unlike most other auto-active *R* gene alleles, RRS1^SLH1^ carries a single amino acid insertion in the WRKY-DNA binding domain that reduces its DNA-binding affinity [30]. To address the role of RRS1 in transcriptional activation or repression, we tested whether RRS1^SLH1^-induced transcription changes overlap with AvrRps4- or PopP2-triggered transcription changes. Based on previously reported expression profiling data and the present study, we propose that the genes whose transcripts were differentially regulated by RRS1^SLH1^, and by AvrRps4 and PopP2 are directly regulated by RRS1 upon Avr detection. As exons 6 and 7 of RRS1^SLH1^ show reduced binding to a W-box *in vitro*, RRS1 may act as a transcriptional repressor of plant immunity, or at least as a repressor of RPS4 function, and this repression may be relieved upon Avr perception [30]. However, RRS1 could act both as repressor and activator of defense gene transcription, as has been found for other plant transcription factors [50]. Loss of RRS1-DNA binding may be part of the activation of defense transcription, but paradoxically, *rrs1* knockout lines do not show enhanced immunity.

Identification of RPS4 mutant alleles among the *SUSHI* mutations was unexpected, as we had anticipated that RRS1 might act downstream of RPS4 to regulate defense gene transcription directly. Notably, it would have been difficult to recover recombinants between RRS1^SLH1^ and an adjacent mutant allele of RPS4, so without a genetic screen, this discovery might not have been made. Based on the genetic requirement of RPS4 for RRS1^SLH1^-induced defense gene transcription, we now hypothesize that RPS4 is required to form a functional immune receptor complex with RRS1. This hypothesis is further supported by the fact that RPS4 and RRS1 interact with each other, in part but not solely by forming a TIR-TIR domain heterodimer [43]. In addition, the requirement of a functional P-loop motif for RPS4 but not for RRS1 function suggests that RPS4 contributes to defense activation by providing ATP-dependent conversion of a repressive immune receptor complex to an activated state. PopP2 interacts with RRS1 [46], as does AvrRps4 [43]. We hypothesize that RPS4 activates defense upon recognition of perturbations in RRS1 by effectors, and that RRS1^SLH1^ mimics the results of effector action upon RRS1. Can this be reconciled with the observation that a *35S:RPS4* constitutive defense phenotype partially requires RRS1 [51]? Conceivably, RRS1 might also play a chaperone-like role in facilitating conversion of RPS4 from an inactive to an active form, and RRS1^SLH1^ has enhanced activity in facilitating this conversion.

The TIR domain of RPS4 induces cell death when transiently overexpressed in tobacco. Several amino acid residues were shown to be required for RPS4 TIR domain auto-activity [42]. Among the 33 single amino acid polymorphisms of RPS4 that we identified in *sushi* mutants, two residues, R28 and E88, were previously implicated as being required for RPS4 TIR domain-induced auto-activity in tobacco. R28H and E88K mutations are unlikely to alter the overall structure of RPS4 TIR domain, judging from the crystal structure of RPS4/RRS1 TIR domain heterodimer [43]. A study on RPS4 natural variants identified Y950 as an important residue for function as a susceptible RLD allele of RPS4 carries a Y950H mutation, and a Y950H substitution in the functional L*er* allele of RPS4 abolishes its AvrRps4-recognition capability [24]. Interestingly, we identified several mutations (S914F, G952E and G997E) in this C-terminal domain (CTD) of RPS4. Although the function of the RPS4 CTD remains unclear, it appears to be important for immune signaling. Conceivably, the *sushi*-mutated residues found in the TIR domain (R28, E88, P105L and G120R) and in the CTD (S914F, G952E, and G997E) are involved in the interaction with RRS1 or other yet unknown partner(s).

AvrRps4 and PopP2 interact directly with RRS1 [43], [46]. Conceivably, after interaction of AvrRps4 or PopP2 with RRS1, dissociation of the activated RPS4/RRS1 immune complex from target DNA induces RPS4 P-loop-dependent de-repression/activation of defense gene transcription, perhaps *via* WRKY18 and WRKY40 [47]. There may be multiple WRKY transcription factors that can replace the transcriptional repression function of RRS1, but not its Avr-recognition function. However, the Ws-2 RRS1^SLH1^ allele may make additional contributions to assembling a defense-activating complex beyond vacating W-boxes.

An intriguing feature of RRS1 is that it is the only known recessive NB-LRR-encoding *R* gene. Consistent with this observation, the *slh1* mutation is also recessive. We were able to recapitulate this feature by transiently co-expressing RRS1 with RPS4 and RRS1^SLH1^ and suppressing RPS4/RRS1^SLH1^-triggered HR. This suppression is abolished if the RRS1-R carries a mutation in its P-loop motif. Intriguingly, this result suggests that the RRS1-R P-loop is not required for RPS4-dependent HR activation, but potentiates assembly of an inactive, poised complex. Thus, we suggest that the recessive nature of RRS1 in the Col-0(S)/Nd-0(R) or Col-0(S)/Ws-2(R) cross is the result of the Col-0 allele encoding a protein that can interfere *in trans* with PopP2 responsiveness and thus acts as a “poison subunit”.

There are nine TIR-NB-LRR gene pairs reported in the Arabidopsis Col-0 genome [26]. It is important to better understand how paired R proteins have evolved and recognize effectors. It is interesting to note that all three TIR-NB-LRR-WRKY encoding genes (At5g45260, At5g45050 and At4g12020) found in Arabidopsis are paired with TIR-NB-LRR genes [26]. This suggests that at least some other paired R proteins may function cooperatively in the nucleus by directly regulating transcriptional processes.

In conclusion, the deployment of a *Pseudomonas* T3S delivery of PopP2 allowed a detailed comparison of AvrRps4- and PopP2-triggered RPS4- and RRS1-dependent transcriptional regulation. We found that an auto-active allele of the TIR-NB-LRR-WRKY protein RRS1, RRS1^SLH1^, induces immune responses comparable to Avr-triggered immunity. The *suppressor of* slh1 *immunity* screening enabled us to uncover the critical role of RPS4 in RRS1^SLH1^-mediated defense activation. Furthermore, we defined additional properties of RPS4 and RRS1 that are essential for function, and these results significantly enhance our understanding of NB-LRR protein function in plants.

## Materials and Methods

### Plant materials and growth conditions

Arabidopsis plants were grown in short day conditions (10 h light/14 h dark) at 21°C or 28°C. *Nicotiana benthamiana* and *Nicotiana tabacum* ‘Petit Gerard’ plants were grown in long day conditions (16 h light/8 h dark) at 24°C. No-0 and *slh1* are described in [30]; Ws *rrs1-1* and Ws *rrs1-1 rps4-21* are described in [26].

### Plasmid constructions

To create pEDV6 (gateway destination variant of pEDV3), the nucleotide sequence encoding the HA epitope tag was inserted at *Sal*I site of pEDV3 [33] that resulted in AvrRps4N(1-137aa):HA:*Cla*I:*BamH*I (pEDV5). Subsequently, pEDV5 was digested with *Cla*I and B*amH*I, treated with T4 DNA-polymerase to generate blunt ends and ligated with *EcoR*V digested Gateway reading frame cassette B (RFB) (Invitrogen) to create pEDV6. Construction of pBBR1MCS-5:*avrRps4* was described previously [35]. The NES- or NLS-tagged avrRps4 variants were kindly provided by Jane Parker laboratory and the cloning procedure was described previously [8]. To generate pEDV6:*popP2* variants, full-length or truncated *popP2* fragments were amplified from *Ralstonia solanacearum* genomic DNA by polymerase chain reaction and cloned in the Gateway entry vector, pCR8 (Invitrogen). Introduction of *popP2* fragments in pEDV6 was performed according to manufacturer's instructions by using LR recombinase II (Invitrogen). The pBin19:RPS4:HA construct was described previously [52]. To obtain C-terminally GFP tagged AvrRps4 or PopP2 variants, *avrRps4* or *popP2* coding regions were PCR amplified and cloned at *Cla*I and *BamH*I sites of EpiGreenB5:GFP. Construction of 35S:RRS1:His-Flag is described in [43]. Wild type and mutant variants of AvrRps4 and PopP2 were PCR amplified from previously reported plasmid constructs [35], [53]. The resulting PCR fragments were cloned in pCR8 (Invitrogen) and correct sequences were confirmed. These pCR8 constructs were used for LR reaction with the Gateway destination vector pK7FWG2 (35S promoter and C-GFP) to generate C-terminally GFP-tagged AvrRps4 and PopP2 variants. Wild type and SH-AA mutant variants of RPS4-HA and RRS1-His-Flag are described in [43]. Introduction of SLH1 and SUSHI mutations in RRS1 and RPS4, respectively, was achieved by using Quikchange II XL site-directed mutagenesis kit (Agilent). The C-terminally GFP-tagged RPS4 constructs were generated by inserting *Cla*I/*BamH*I digested RPS4 in EpiGreenB5-GFP-WT/NES/NLS.

### Bacterial strains, culture conditions and manipulations

*Escherichia coli* DH5α was used for maintaining plasmid constructs and bacterial conjugation. For hypersensitive response assay and *in planta* bacterial growth assay, *Pseudomonas fluorescens* Pf0-1(T3S) and *Pseudomonas syringae* pv. *tomato* (*Pto*) DC3000 strains were used, respectively. To introduce various constructs carrying *avrRps4*, *popP2* or *hopA1* in Pf0-1(T3S) and *Pto* DC3000, standard triparental mating method was used by using *E. coli* HB101 (pRK2013) as a helper strain as previously described [33]. For transformation of *Agrobacterium tumefaciens* strain AGL1, standard electroporation method was used.

### Plant pathology experiments

For hypersensitive response assay, freshly grown Pf0-1 (T3S) strains on King's B agar plates containing appropriate antibiotics were harvested in 10 mM MgCl_2_. The final concentration of bacterial suspensions was adjusted to *A*_600_ = 0.2. Leaves of five week-old Arabidopsis plants were hand-infiltrated by using 1 mL needless syringes and kept 20–24 h further for symptom development. For ion leakage assays, leaf discs were sampled at 0.5 hpi, floated on water for 30 minutes (with gentle shaking at room temperature) and transferred to fresh water (1 hpi sample). Ion leakage was measured at 24 hpi using a conductivity meter. For *in planta* bacterial growth assays, *Pto* DC3000 strains were grown and harvested as for Pf0-1(T3S). Leaves of five week-old Arabidopsis plants were hand-infiltrated with bacterial suspensions (*A*_600_ = 0.001) by using 1 mL needless syringes and kept 3–4 days further before sampling. Infected leaf samples were ground in 10 mM MgCl_2_, serially diluted, spotted on L agar plates containing appropriate antibiotics and kept at 28°C for 2 days before counting colonies to estimate bacterial population in infected leaves.

### Agrobacterium-mediated transient transformation of *Nicotiana benthamiana* and *Nicotiana tabacum*

*Agrobacterium tumefaciens* AGL1 strains carrying the different constructs were grown in liquid L-medium supplemented with adequate antibiotics for 24 h. Cells were harvested by centrifugation and re-suspended at OD_600_ 0.5 in infiltration medium (10 mM MgCl_2_, 10 mM MES pH 5.6). For co-expression, bacterial suspensions were mixed in 1∶1 ratio before infiltration with needleless syringes in 5 week-old *N. benthamiana* or *N. tabacum* leaves. Tobacco hypersensitive response was generally observed and photographed 2 to 3 days after infiltration.

### EXPRSS library, Illumina sequencing and transcriptional profiling analysis

EXPRSS tag-seq cDNA library construction and data analysis was carried out as described previously [38]. The sequence data presented in this publication have been deposited in NCBI's Gene Expression Omnibus [54] and are accessible through GEO Series accession number GSE48247 and GSE51116. Tag to gene associations were carried out using uniquely mapped reads, with the considerations described previously [38]. Bowtie v0.12.8 [55] was used to map short reads to TAIR10 genome and Novoalign v2.08.03 (http://www.novocraft.com/) was used to align remaining reads to TAIR10 cDNA sequences. Differential gene expression analysis was performed using the R statistical language (v2.11.1) with the Bioconductor package [56], edgeR v1.6.15 [57] with the exact negative binomial test using tagwise dispersion and selected genes with false discovery rate (FDR) <0.01. From RNA-seq data for avrRps4 on Col-0 [29], uniquely mapped read counts to genes were used for reanalysis using edgeR and selected gene with FDR <0.05.

Microarray data files from *Pto* DC3000 (AvrRps4) infiltration (Array Express E-MEXP-546, [28]) and Interaction of *Arabidopsis thaliana* and *Ralstonia solanacearum* (NASCARRAYS-447, [40]) were used. Data analysis was performed using the R statistical language as described previously [38], [58]. Differentially expressed genes were identified using the rank products method with FDR <0.05 [59]. As *Pto* DC3000 (AvrRps4) data has only one replicate, differential expression analysis was carried out with untreated and MgCl_2_ infiltrated 3 hpi samples as controls and compared against 3 and 6 hpi of avrRps4 and 6 hpi of MgCl_2_. For GMI1000/GMI1000ΔPopP2 data, only Nd-1 samples were used.

### Marker gene expression analysis by qRT-PCR

Total RNAs were extracted from 4 to 5 week-old Arabidopsis plants using the TRI reagent (Invitrogen) according to the manufacturer's instructions. First-strand cDNA was synthesized from 5 µg RNA using SuperScriptII Reverse Transcriptase (Invitrogen) and an oligo(dT) primer, according to the manufacturer's instructions. cDNA was amplified in triplicate by quantitative PCR using SYBR Green JumpStart Taq ReadyMix (Sigma) and the CFX96 Thermal Cycler (Bio-Rad). The relative expression values were determined using the comparative Ct method and *Ef1α* (At5g60390) as reference. Primers used for quantitative PCR are described in Table S7.

### *slh1* genotyping and candidate genes coding region sequencing

The presence of the *slh1* mutation in *sushi* M3 generation and F1 individuals resulting from the genetic cross with *rrs1-1* or *rrs1-1 rps4-21* was assessed using the CAPS marker described in [30]. For sequencing of candidate genes on *sushi* mutants genomic DNA, 10, 6, 4 and 4 couples of primers respectively were used to amplify regions of *RRS1*, *RPS4, EDS16* and *EDS5* coding sequence (see Table S7). PCR products were purified on Sepharose and sequences were analyzed using the Vector NTI assembly software (Invitrogen).

### Protein extraction, immunoprecipitation and immunoblotting

Protein samples were prepared from *N. benthamiana* 48 h after *Agrobacterium*-mediated transformation. One infiltrated leaf was harvested and ground in liquid nitrogen. Total proteins were extracted in GTEN buffer (10% glycerol, 100 mM Tris-HCl pH 7.5, 1 mM EDTA, 150 mM NaCl) supplemented extemporaneously with 5 mM DTT, 1% (vol/vol) plant protease inhibitor cocktail (Sigma) and 0.2% (vol/vol) Nonidet P-40. Lysates were centrifuged for 15 min at 5,000 *g* at 4°C and aliquots of filtered supernatants were used as input samples. Immunoprecipitations were conducted on 1.5 mL of filtered extract incubated for 2 h at 4°C under gentle agitation in presence of 20 µL anti-FLAG M2 or EZview anti-HA affinity gel (Sigma). Antibodies-coupled agarose beads were collected and washed three times in GTEN buffer, re-suspended in SDS-loading buffer and denatured 10 min at 96°C. Proteins were separated by SDS-PAGE and analyzed by immunoblotting using anti-FLAG M2-HRP, anti-GFP-HRP or anti-HA-HRP conjugated antibodies (Sigma, Santa Cruz and Roche respectively).

## Supporting Information

Figure S1 PopP2^149–488^ triggers Arabidopsis immunity when delivered from *Pseudomonas*. (*A*) Construction of pEDV5 and pEDV6 and functional analysis of N-terminally truncated PopP2 variants. (*B*) Schematic presentation of PopP2 protein. The numbers indicate the corresponding amino acids of full-length PopP2. Cys321 is required for acetyltransferase activity [36]. NLS: nuclear localization signal. (*C*) Hypersensitive response (HR) assay in Ws-2. *Pseudomonas fluorescens* Pf0-1(T3S) strains expressing wild type or N-terminally truncated PopP2 variants were used for inoculating leaves of five week-old Ws-2 plants. The photograph was taken at 24 hpi. Red arrows indicate the leaves showing HR. (*D*) *Pseudomonas syringae* pv. *tomato* (*Pto*) DC3000 strains expressing indicated PopP2 variants were used for inoculating leaves of five week-old Ws-2 plants. The results presented are the mean and standard error of the number of bacterial colonies recovered. Means labeled with the same letter are not statistically different at the 5% confidence level based on Tukey's test.
(TIF)

Figure S2 Nuclear localization of PopP2 and AvrRps4 is sufficient to trigger RPS4/RRS1-dependent hypersensitive response and immunity in Arabidopsis. (*A*) Hypersensitive response (HR) assay in wild type and transgenic RLD or Col-0 expressing RPS4^Ler^ or RRS1^Ws-2^, respectively. Leaves of five week-old Arabidopsis were infiltrated with Pf0-1(T3S) expressing AvrRps4N:PopP2^149–488^ variants. The photograph was taken at 24 hpi. The red asterisks indicate the leaves showing HR. (*B*) PopP2^NLS^ triggers elevated ion leakage level in Ws-2. Infection conditions were same as in (*A*). (*C*) Nuclear localization of PopP2 is necessary and sufficient to trigger immunity. *Pto* DC3000 expressing AvrRps4N:PopP2^149–488^ variants were used for infection of five week-old wild type Ws-2 plants. Infected leaf samples were taken at 4 dpi to measure bacterial numbers. Means labelled with the same letter are not statistically different at the 5% confidence level based on Tukey's test. (*D*) Marker gene expression analysis by qRT-PCR. (*E*) Nuclear localization of PopP2 is required to trigger HR in Ws-2. Experimental conditions were the same as in (*A*). NES and NLS indicate nuclear export signal and nuclear localization signal, respectively.
(TIF)

Figure S3 Expression analysis of PopP2 variants in Pf0-1 (T3S). *Pseudomonas fluorescens* Pf0-1(T3S) strains carrying the indicated *pEDV6:PopP2^149–488^* variant was freshly prepared and used for infection of 4-weeks old *Nicotiana benthamiana* leaves (*A*_600_ = 2.0). Samples for total protein extraction were taken at 10 hpi. Experimental procedures used in this study were identical to Williams et al. [43].
(TIF)

Figure S4 Verification of gene induction during PopP2-triggered RRS1-dependent immunity. RRS1- and PopP2-dependent gene expression was verified with qRT-PCR of *EDS5*, *NudT6*, *WRKY18* and *WRKY40* on the cDNA used for Illumina libraries. Induction of *EDS5* and *NudT6* was primarily due to RPS4/RRS1-R recognition of PopP2, while expression of *WRKY18* and *WRKY40* appears mainly due to PTI. Expression values represent the mean from three biological replicates and error bars indicate the standard error of the mean.
(TIF)

Figure S5 Transcriptional profiling of PopP2-infected plants shows significant overlap with AvrRps4-regulated genes. (*A*) Venn diagram presenting the overlap between PopP2/Ws-2 (FDR <0.001), AvrRps4/Col-0 (FDR <0.05), AvrRps4/Ws-2 (FDR <0.05) and GMI1000/Nd-1 (FDR <0.05) differentially regulated genes. (*B*) Pairwise comparison of differential gene expression among the four experiments. Numbers of genes unique to each data set are presented on the diagonal (**^a^**). Overlap is represented as a number common between pairwise comparisons, below the diagonal (**^c^**) and hypergeometric probability values of the overlap are represented above the diagonal (**^b^**).
(TIF)

Figure S6 Defense marker gene expression is abolished in fully rescued *sushi* mutants. Wild type No-0, *slh1* and *sushi* mutant lines were grown at 28°C for four weeks then plants were shifted at 21°C for 24 h. Transcript accumulation of defense marker gene was determined by qRT-PCR and is presented relative to No-0 before temperature shift.
(TIF)

Figure S7 Heterozygosity at *RRS1* locus in *sushi* × Ws *rrs1-1* and *sushi* × *rrs1-1 rps4-21* F1 individuals. *slh1* CAPS marker [30] was used for PCR amplification using the genomic DNA from individual *sushi* × Ws *rrs1-1* and *rrs1-1 rps4-21* F1 shown in Figure 6B, and digested with *Dde*I. Size (bp) of the uncleaved (*RRS1^SLH1^*_352) and cleaved (*RRS1^WT^*_223 and 129) product is shown on the left.
(TIF)

Figure S8 Recapitulation of RPS4/RRS1-dependent recognition of AvrRps4 and PopP2 in tobacco. (*A*) Recognition of AvrRps4 or PopP2 in tobacco requires previously shown properties. The photograph was taken 3 days after agroinfiltration. (*B*) qRT-PCR analysis of selected defence genes in response to PopP2 recognition in tobacco. Agroinfiltrated leaf samples were taken at indicated times for total RNA extraction. Expression results are mean from two biological replicates and error bars indicate standard error of the mean.
(TIF)

Figure S9 RRS1 interact with RPS4 variants. Variants of RRS1-His-Flag (RRS1^SLH1^, RRS1^SH-AA^, RRS1^SLH1/SH-AA^) and RPS4-HA (RPS4^K242A^, RPS4^SH-AA^, RPS4^sushi^) were transiently expressed in *Nicotiana benthamiana* and subjected to immunoprecipitation. RPS4^sushi^ variants interact with (*A*) RRS1^WT^ or (*B*) RRS1^SLH1^. (*C*) Mutations in RPS4 or RRS1 that affect the Avr-recognition capacity do not alter the full-length RPS4-RRS1 interaction in co-immunoprecipitation assays.
(TIF)

Figure S10 RPS4^sushi^ do not have a dominant negative effect on RPS4^WT^. RPS4^sushi^ variants cannot interfere with RPS4^WT^/RRS1^SLH1^-mediated cell death in tobacco. Photographs were taken 3 days after agroinfiltration.
(TIF)

Figure S11 RPS4^TIR^ *sushi* mutant (M3), wild type No-0 and *slh1* plants were grown at 21°C for 25 days. (*A*) Plant morphology. (*B*) qRT-PCR analysis of selected RRS1^SLH1^-regulated genes. Transcript accumulation is presented relative to No-0.
(TIF)

Figure S12 Immunoblot analysis of transiently expressed proteins. Leaf samples infiltrated with *Agrobacterium* strains were taken at 2 dpi. Total protein extracts were used for immunoblot analysis. (*A*) Expression of GFP-tagged AvrRps4 or PopP2^149–488^ proteins. (*B*) Expression of full-length RPS4^K242A^ and RPS4^SH-AA^ variants is comparable to wild type RPS4-HA. (*C*) Expression of variants of full-length RRS1-His-Flag. (*D*) Expression of RPS4^TIR^-GFP variants carrying *SUSHI* mutations. (E) Expression of full-length RPS4-HA variants carrying TIR *SUSHI* mutations.
(TIF)

Table S1 Details of genes differentially expressed in the RRS1-PopP2 time course.
(XLSX)

Table S2 Details of GO enrichment analysis for genes differentially expressed during the RRS1-PopP2 time course.
(XLSX)

Table S3 Mean expression (in Tag counts per million) of genes differentially induced in the RRS1-PopP2 time course.
(XLSX)

Table S4 Details of genes differentially expressed in the Col-0-AvrRps4 [29], Ws-2-AvrRps4 [28] and Nd-1-GMI1000 time course [40].
(XLSX)

Table S5 Details of gene differentially expressed during the *slh1*/No-0 temperature shift time course.
(XLSX)

Table S6 Candidate gene mutations found in *sushi*.
(DOCX)

Table S7 Primers used in this study.
(DOCX)
