# Supplementary figures and images for: The Nuclear Immune Receptor RPS4 Is Required for RRS1SLH1-Dependent Constitutive Defense Activation in Arabidopsis thaliana
Source: PLoS Genet. 2014 Oct 23;10(10):e1004655. doi: 10.1371/journal.pgen.1004655 (PMC4207616; doi:10.1371/journal.pgen.1004655)

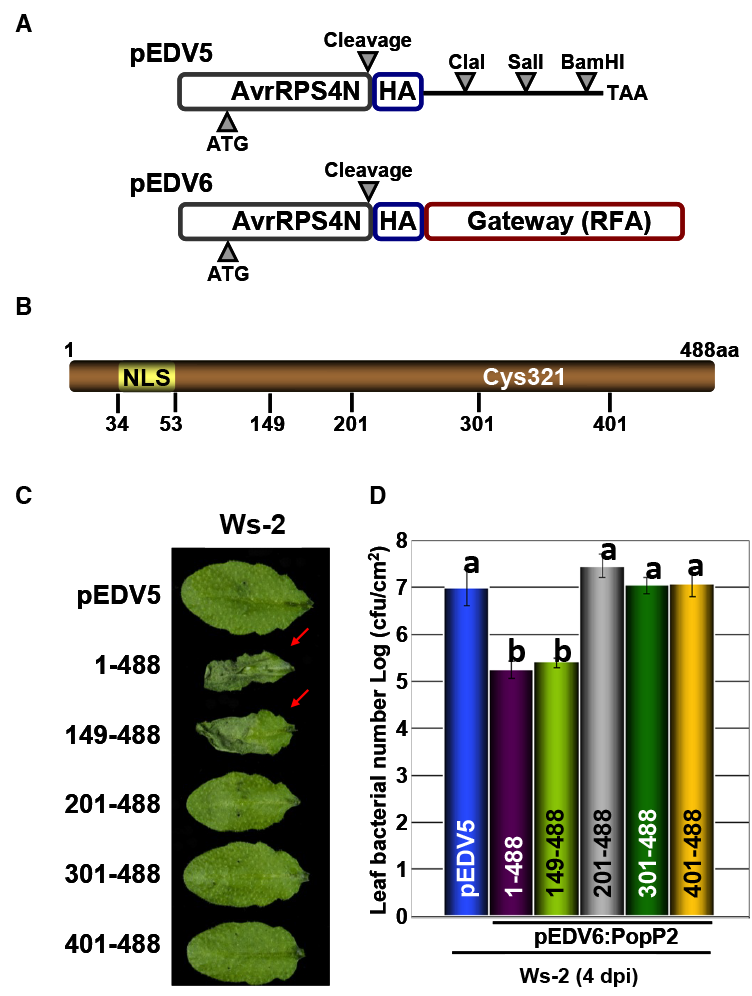

Supplement: Figure S1 — PopP2149–488 triggers Arabidopsis immunity when delivered from Pseudomonas. (A) Construction of pEDV5 and pEDV6 and functional analysis of N-terminally truncated PopP2 variants. (B) Schematic presentation of PopP2 protein. The numbers indicate the corresponding amino acids of full-length PopP2. Cys321 is required for acetyltransferase activity [36]. NLS: nuclear localization signal. (C) Hypersensitive response (HR) assay in Ws-2. Pseudomonas fluorescens Pf0-1(T3S) strains expressing wild type or N-terminally truncated PopP2 variants were used for inoculating leaves of five week-old Ws-2 plants. The photograph was taken at 24 hpi. Red arrows indicate the leaves showing HR. (D) Pseudomonas syringae pv. tomato (Pto) DC3000 strains expressing indicated PopP2 variants were used for inoculating leaves of five week-old Ws-2 plants. The results presented are the mean and standard error of the number of bacterial colonies recovered. Means labeled with the same letter are not statistically different at the 5% confidence level based on Tukey's test. (TIF) [file pgen.1004655.s001.tif]

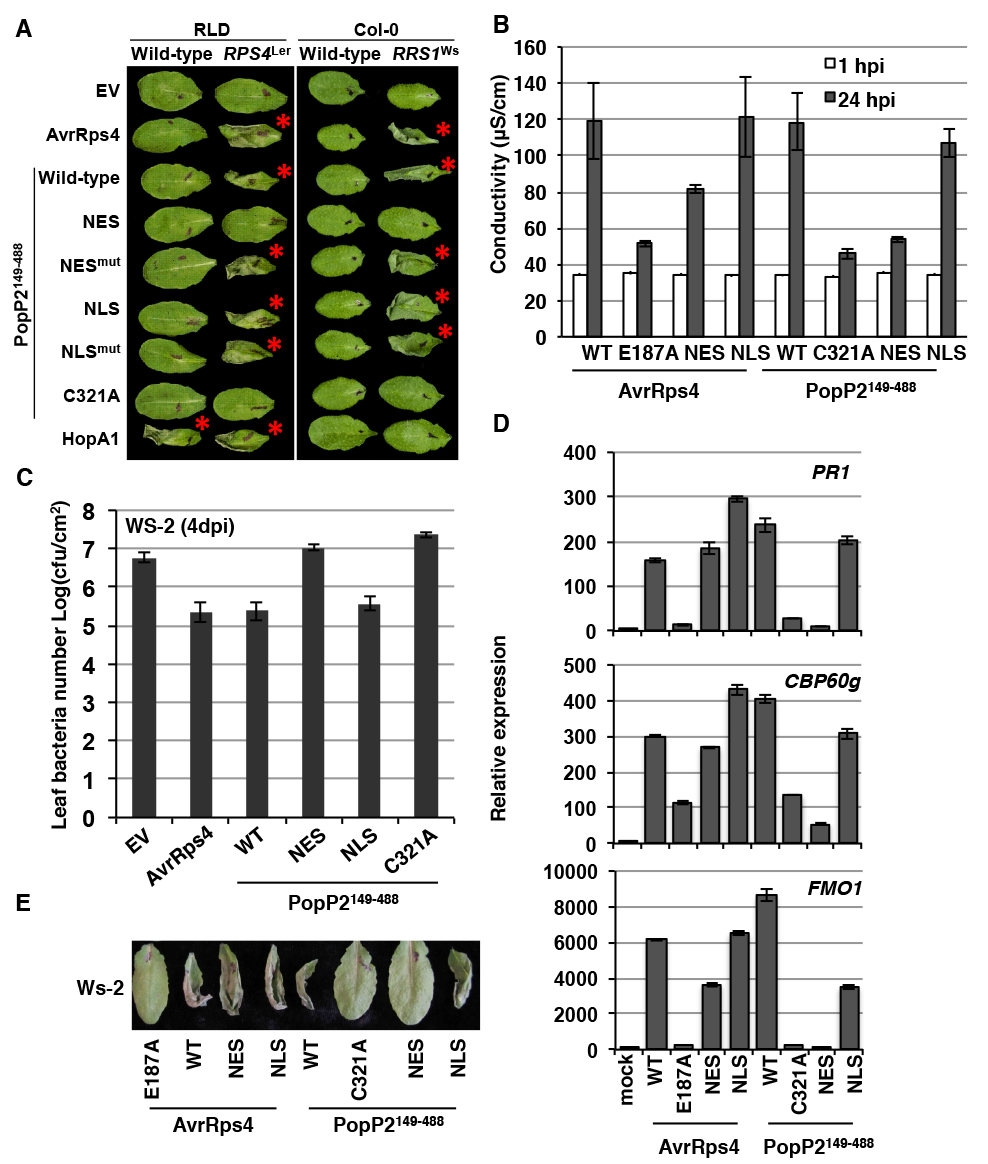

Supplement: Figure S2 — Nuclear localization of PopP2 and AvrRps4 is sufficient to trigger RPS4/RRS1-dependent hypersensitive response and immunity in Arabidopsis. (A) Hypersensitive response (HR) assay in wild type and transgenic RLD or Col-0 expressing RPS4Ler or RRS1Ws-2, respectively. Leaves of five week-old Arabidopsis were infiltrated with Pf0-1(T3S) expressing AvrRps4N:PopP2149–488 variants. The photograph was taken at 24 hpi. The red asterisks indicate the leaves showing HR. (B) PopP2NLS triggers elevated ion leakage level in Ws-2. Infection conditions were same as in (A). (C) Nuclear localization of PopP2 is necessary and sufficient to trigger immunity. Pto DC3000 expressing AvrRps4N:PopP2149–488 variants were used for infection of five week-old wild type Ws-2 plants. Infected leaf samples were taken at 4 dpi to measure bacterial numbers. Means labelled with the same letter are not statistically different at the 5% confidence level based on Tukey's test. (D) Marker gene expression analysis by qRT-PCR. (E) Nuclear localization of PopP2 is required to trigger HR in Ws-2. Experimental conditions were the same as in (A). NES and NLS indicate nuclear export signal and nuclear localization signal, respectively. (TIF) [file pgen.1004655.s002.tif]

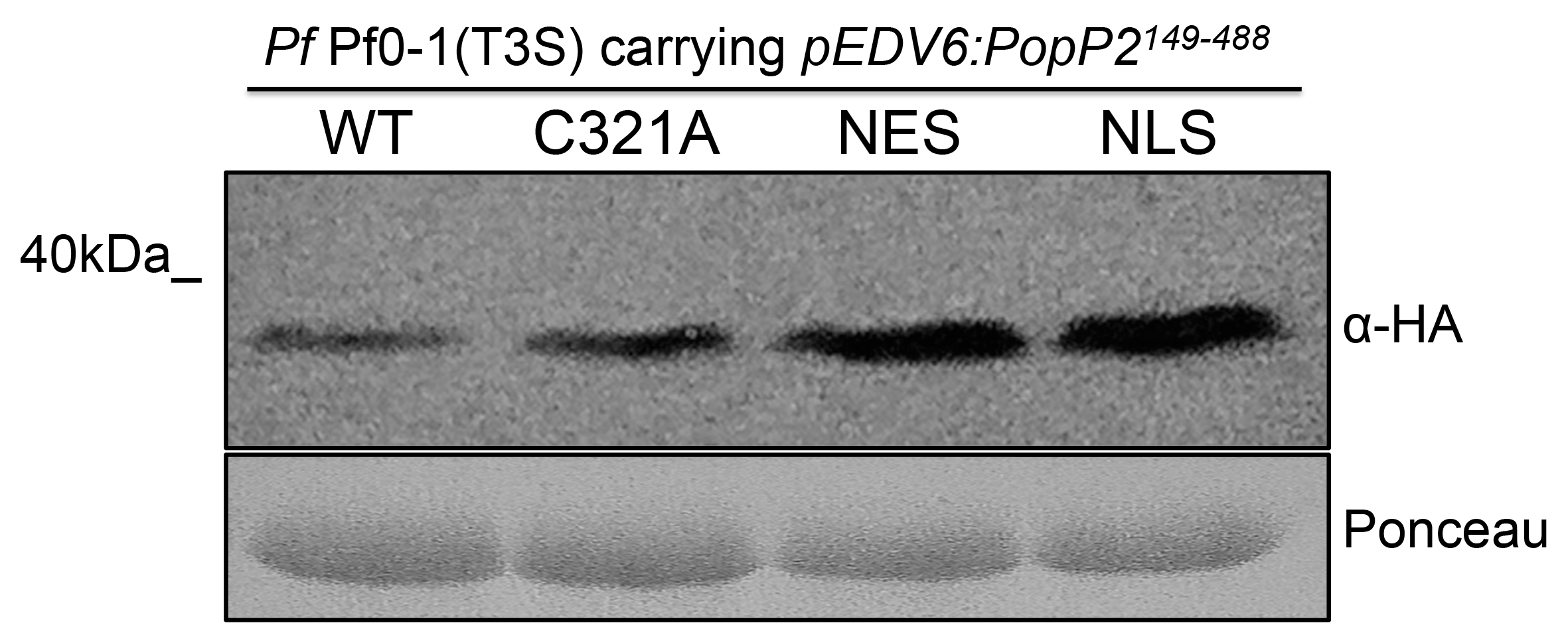

Supplement: Figure S3 — Expression analysis of PopP2 variants in Pf0-1 (T3S). Pseudomonas fluorescens Pf0-1(T3S) strains carrying the indicated pEDV6:PopP2149–488 variant was freshly prepared and used for infection of 4-weeks old Nicotiana benthamiana leaves (A 600 = 2.0). Samples for total protein extraction were taken at 10 hpi. Experimental procedures used in this study were identical to Williams et al. [43]. (TIF) [file pgen.1004655.s003.tif]

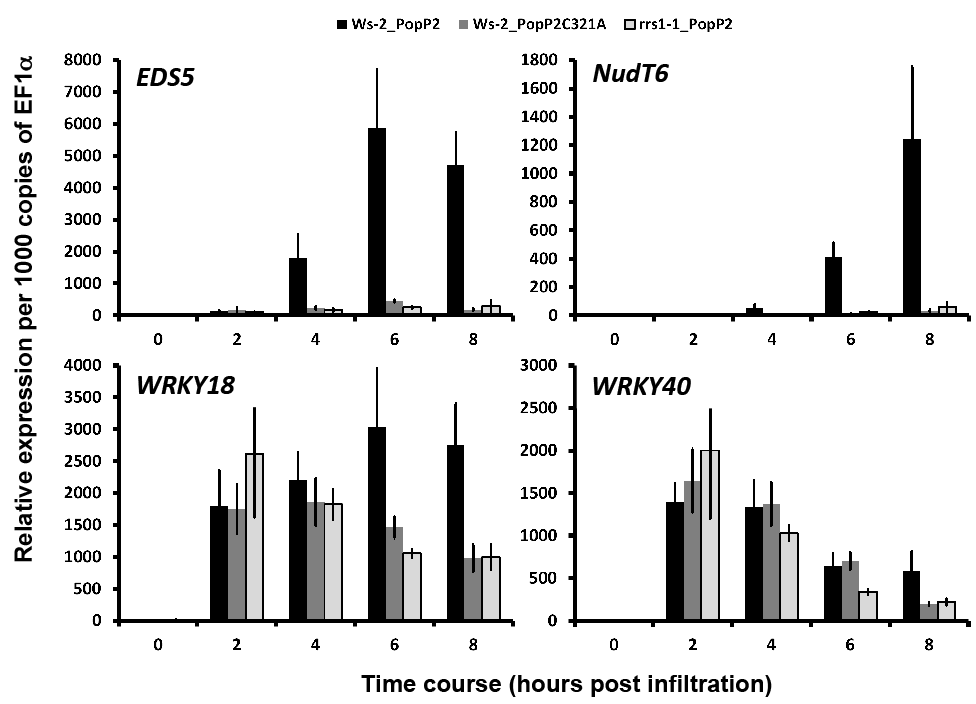

Supplement: Figure S4 — Verification of gene induction during PopP2-triggered RRS1-dependent immunity. RRS1- and PopP2-dependent gene expression was verified with qRT-PCR of EDS5, NudT6, WRKY18 and WRKY40 on the cDNA used for Illumina libraries. Induction of EDS5 and NudT6 was primarily due to RPS4/RRS1-R recognition of PopP2, while expression of WRKY18 and WRKY40 appears mainly due to PTI. Expression values represent the mean from three biological replicates and error bars indicate the standard error of the mean. (TIF) [file pgen.1004655.s004.tif]

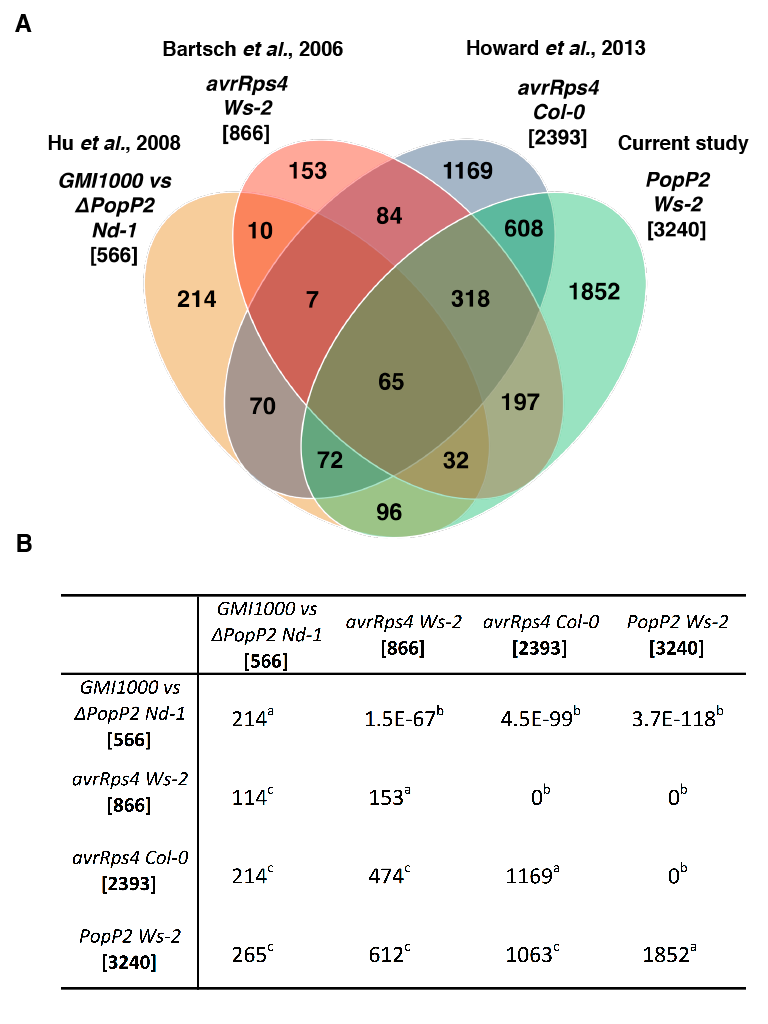

Supplement: Figure S5 — Transcriptional profiling of PopP2-infected plants shows significant overlap with AvrRps4-regulated genes. (A) Venn diagram presenting the overlap between PopP2/Ws-2 (FDR <0.001), AvrRps4/Col-0 (FDR <0.05), AvrRps4/Ws-2 (FDR <0.05) and GMI1000/Nd-1 (FDR <0.05) differentially regulated genes. (B) Pairwise comparison of differential gene expression among the four experiments. Numbers of genes unique to each data set are presented on the diagonal (a). Overlap is represented as a number common between pairwise comparisons, below the diagonal (c) and hypergeometric probability values of the overlap are represented above the diagonal (b). (TIF) [file pgen.1004655.s005.tif]

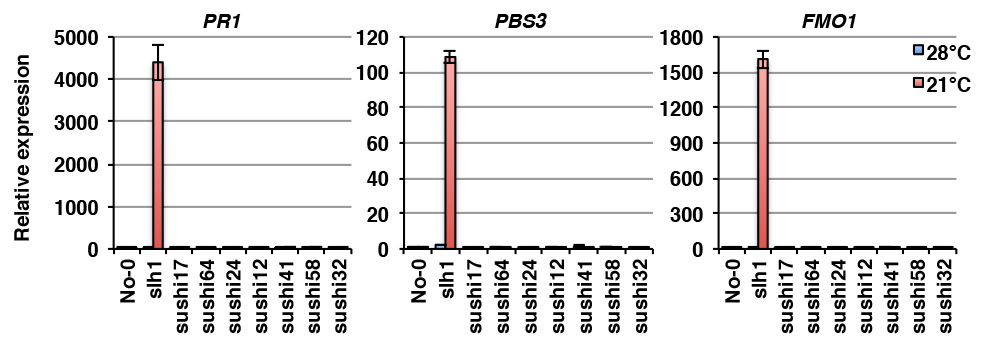

Supplement: Figure S6 — Defense marker gene expression is abolished in fully rescued sushi mutants. Wild type No-0, slh1 and sushi mutant lines were grown at 28°C for four weeks then plants were shifted at 21°C for 24 h. Transcript accumulation of defense marker gene was determined by qRT-PCR and is presented relative to No-0 before temperature shift. (TIF) [file pgen.1004655.s006.tif]

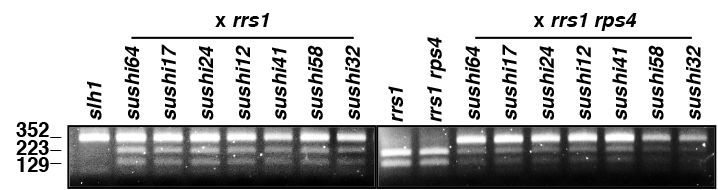

Supplement: Figure S7 — Heterozygosity at RRS1 locus in sushi × Ws rrs1-1 and sushi × rrs1-1 rps4-21 F1 individuals. slh1 CAPS marker [30] was used for PCR amplification using the genomic DNA from individual sushi × Ws rrs1-1 and rrs1-1 rps4-21 F1 shown in Figure 6B, and digested with DdeI. Size (bp) of the uncleaved (RRS1SLH1_352) and cleaved (RRS1WT_223 and 129) product is shown on the left. (TIF) [file pgen.1004655.s007.tif]

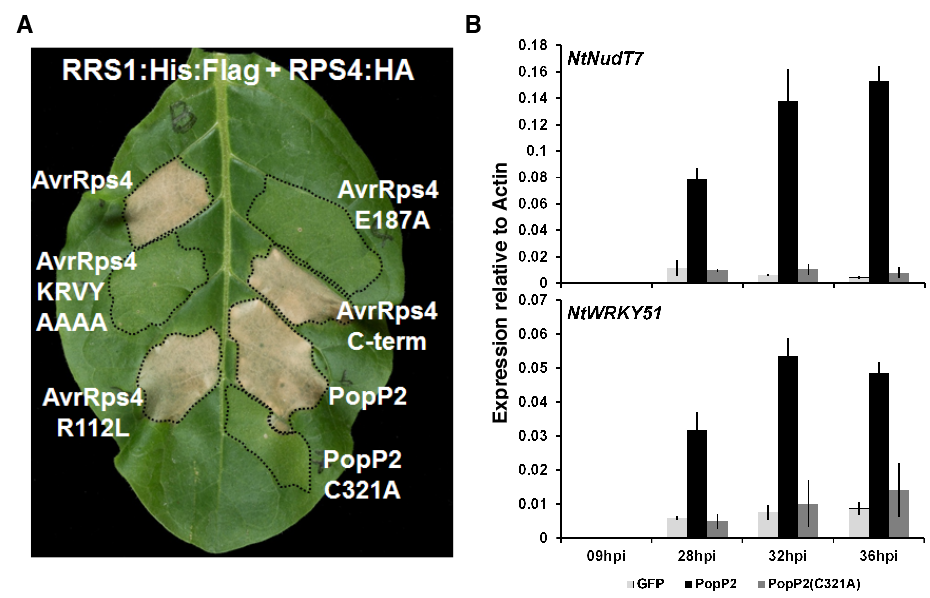

Supplement: Figure S8 — Recapitulation of RPS4/RRS1-dependent recognition of AvrRps4 and PopP2 in tobacco. (A) Recognition of AvrRps4 or PopP2 in tobacco requires previously shown properties. The photograph was taken 3 days after agroinfiltration. (B) qRT-PCR analysis of selected defence genes in response to PopP2 recognition in tobacco. Agroinfiltrated leaf samples were taken at indicated times for total RNA extraction. Expression results are mean from two biological replicates and error bars indicate standard error of the mean. (TIF) [file pgen.1004655.s008.tif]

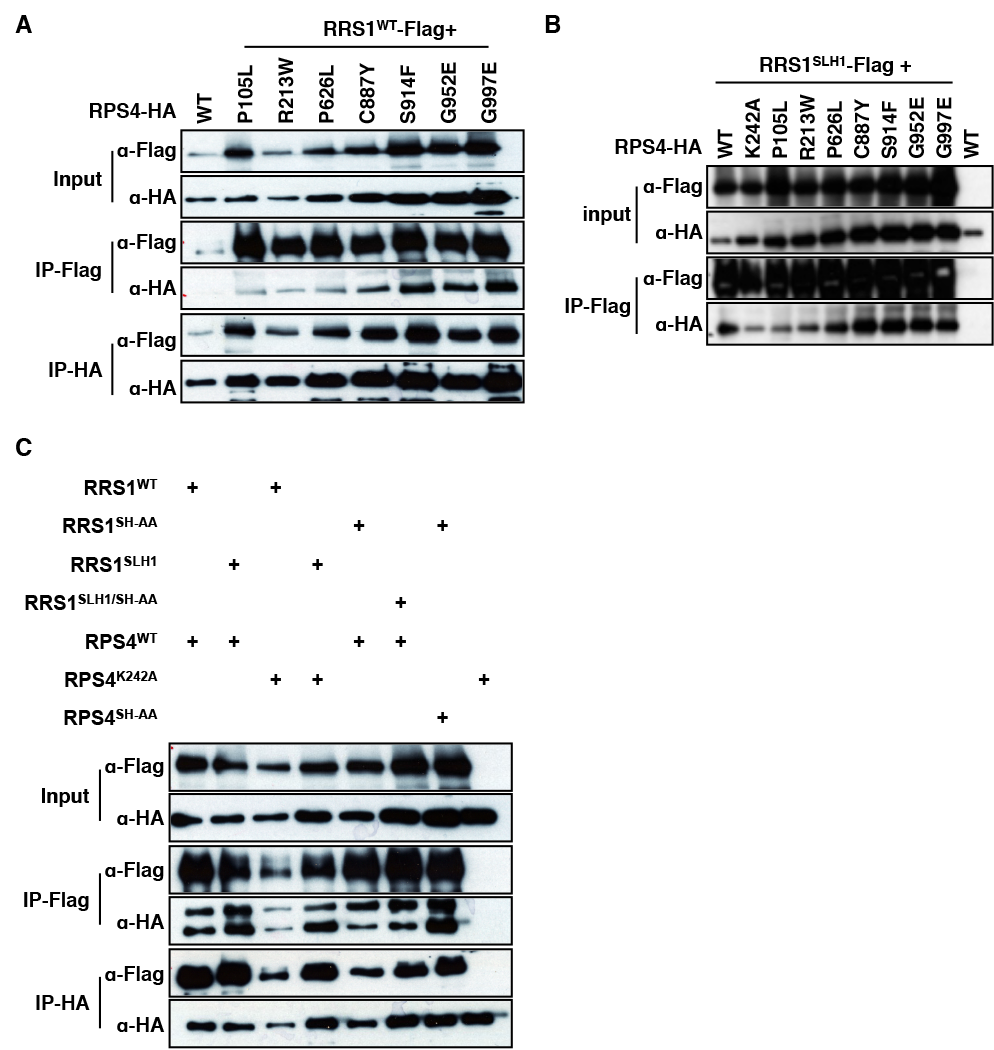

Supplement: Figure S9 — RRS1 interact with RPS4 variants. Variants of RRS1-His-Flag (RRS1SLH1, RRS1SH-AA, RRS1SLH1/SH-AA) and RPS4-HA (RPS4K242A, RPS4SH-AA, RPS4sushi) were transiently expressed in Nicotiana benthamiana and subjected to immunoprecipitation. RPS4sushi variants interact with (A) RRS1WT or (B) RRS1SLH1. (C) Mutations in RPS4 or RRS1 that affect the Avr-recognition capacity do not alter the full-length RPS4-RRS1 interaction in co-immunoprecipitation assays. (TIF) [file pgen.1004655.s009.tif]

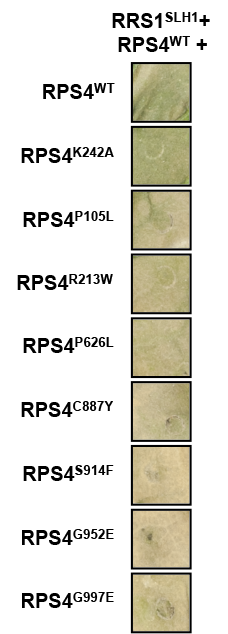

Supplement: Figure S10 — RPS4sushi do not have a dominant negative effect on RPS4WT. RPS4sushi variants cannot interfere with RPS4WT/RRS1SLH1-mediated cell death in tobacco. Photographs were taken 3 days after agroinfiltration. (TIF) [file pgen.1004655.s010.tif]

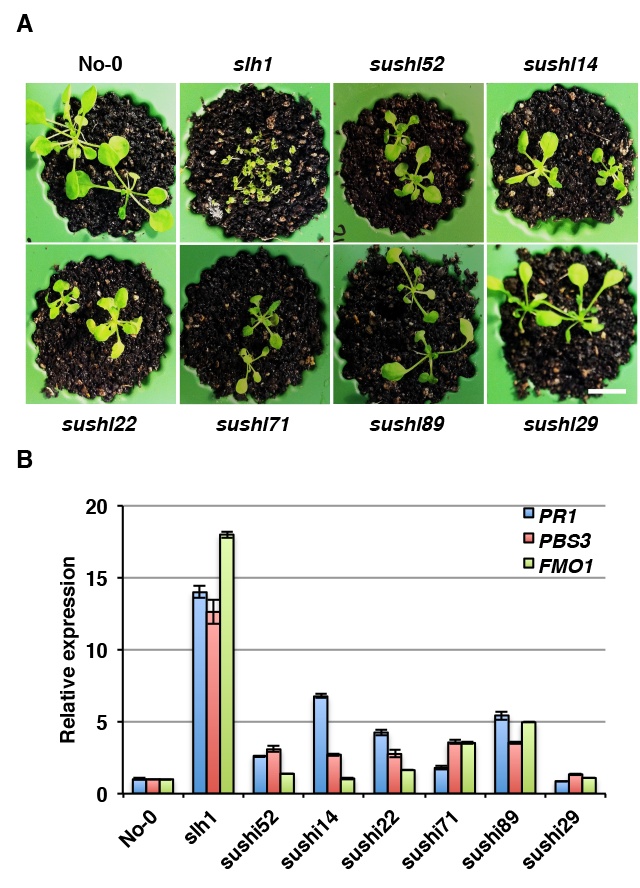

Supplement: Figure S11 — RPS4TIR sushi mutant (M3), wild type No-0 and slh1 plants were grown at 21°C for 25 days. (A) Plant morphology. (B) qRT-PCR analysis of selected RRS1SLH1-regulated genes. Transcript accumulation is presented relative to No-0. (TIF) [file pgen.1004655.s011.tif]

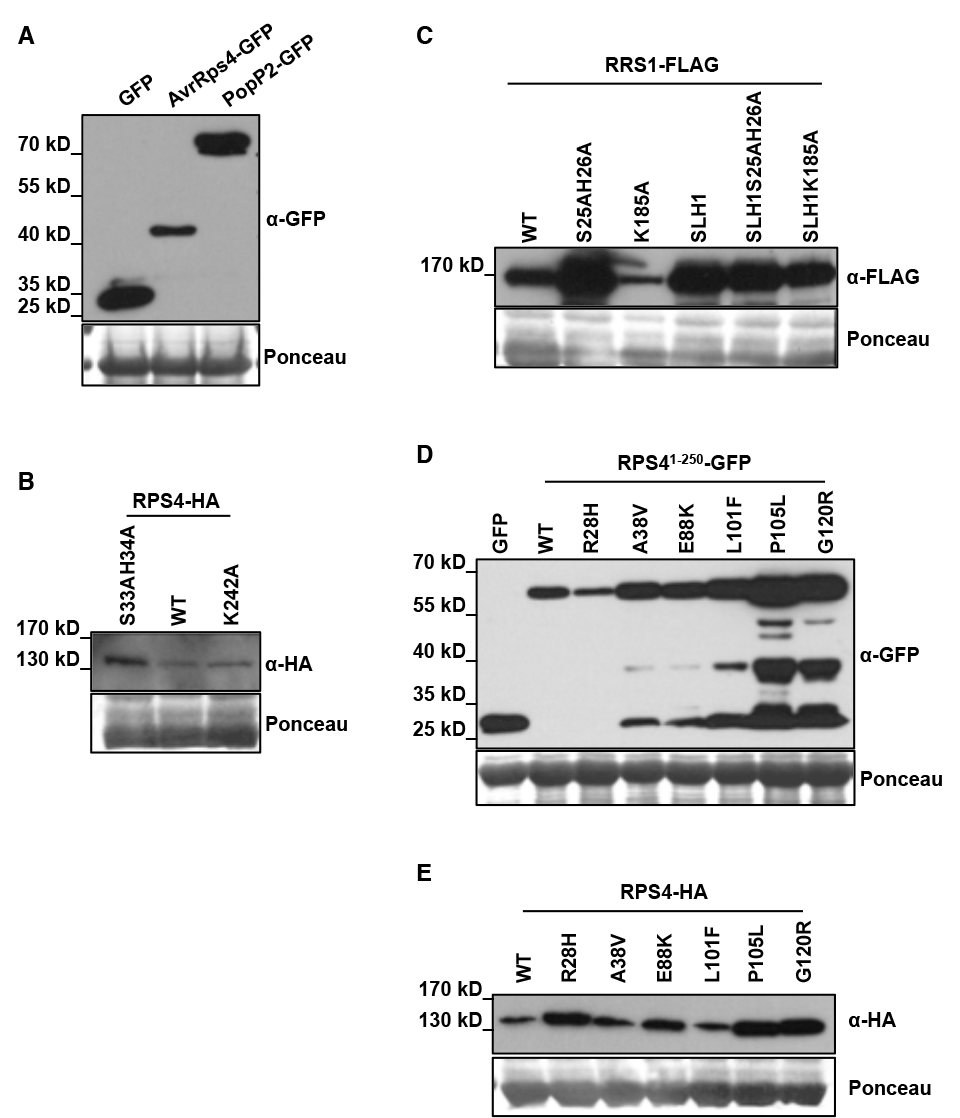

Supplement: Figure S12 — Immunoblot analysis of transiently expressed proteins. Leaf samples infiltrated with Agrobacterium strains were taken at 2 dpi. Total protein extracts were used for immunoblot analysis. (A) Expression of GFP-tagged AvrRps4 or PopP2149–488 proteins. (B) Expression of full-length RPS4K242A and RPS4SH-AA variants is comparable to wild type RPS4-HA. (C) Expression of variants of full-length RRS1-His-Flag. (D) Expression of RPS4TIR-GFP variants carrying SUSHI mutations. (E) Expression of full-length RPS4-HA variants carrying TIR SUSHI mutations. (TIF) [file pgen.1004655.s012.tif]
