# Supplementary material for: The Nuclear Immune Receptor RPS4 Is Required for RRS1SLH1-Dependent Constitutive Defense Activation in Arabidopsis thaliana
Source: PLoS Genet. 2014 Oct 23;10(10):e1004655. doi: 10.1371/journal.pgen.1004655 (PMC4207616; doi:10.1371/journal.pgen.1004655)
Supplement: Table S6 — Candidate gene mutations found in sushi. (DOCX) [file pgen.1004655.s018.docx]

**Table S6.** Candidate gene mutations found in *sushi* mutants.

| ***sushi^[[1]](#footnote-1)^*** | **Gene^[[2]](#footnote-2)^** | **Exon^[[3]](#footnote-3)^** | **Genomic^[[4]](#footnote-4)^** | **Protein^[[5]](#footnote-5)^** |
| --- | --- | --- | --- | --- |
| 84 | RRS1 | 1 | T**G**C>T**A**C | C15Y |
| 40 | RRS1 | 1 | **C**GA>**T**GA | R33* |
| 45 | RRS1 | 1 | C**C**C>C**T**C | P68L |
| 81 | RRS1 | 2 | **C**GA>**T**GA | R151* |
| 11 | RRS1 | 2 | G**G**A>G**A**A | G176E |
| 33 | RRS1 | 2 | T**G**G>T**A**G | W178* |
| 78 | RRS1 | 3 | T**G**G>T**A**G | W441* |
| 26 | RRS1 | 4 | T**G**C>T**A**C | C607Y |
| 23 | RRS1 | 4 | C**C**A>C**T**A | P741L |
| 85 | RRS1 | 4 | **C**AA>**T**AA | Q787* |
| 61 | RRS1 | 4 | **C**GA>**T**GA | R800* |
| 88 | RRS1 | 4 | **C**TT>**T**TT | L814F |
| 50 | RRS1 | 5 | T**C**T>T**T**T | S981F |
| 87 | RRS1 | 7 | T**G**T>T**A**T | C1241Y |
| 52 | RPS4 | 1 | C**G**C>C**A**C | R28H |
| 14 | RPS4 | 1 | G**C**C>G**T**C | A38V |
| 22 | RPS4 | 1 | **G**AG>**A**AG | E88K |
| 71 | RPS4 | 1 | **C**TC>**T**TC | L101F |
| 89 | RPS4 | 1 | C**C**A>C**T**A | P105L |
| 29 | RPS4 | 1 | **G**GA>**A**GA | G120R |
| 72 | RPS4 | 1 | **G**GT>**A**GT | G132S |
| 17 | RPS4 | 2 | **C**GG>**T**GG | R213W |
| 43 | RPS4 | 2 | TG**G**>TG**A** | W253* |
| 30 | RPS4 | 2 | **G**AT>**A**AT | D315N |
| 97 | RPS4 | 2 | TG**G**>TG**A** | W332* |
| 64 | RPS4 | 2 | **C**AA>**T**AA | Q386* |
| 13 | RPS4 | 2 | **C**AG>**T**AG | Q453* |
| 80 | RPS4 | 3 | **G**GT>**A**GT | G559S |
| 10 | RPS4 | 3 | **G**AT>**A**AT | D563N |
| 94 | RPS4 | 3 | A**G**C>A**A**C | S572N |
| 99 | RPS4 | 3 | C**G**C>C**A**C | R575H |
| 82 | RPS4 | 3 | **C**TC>**T**TC | L584F |
| 28 | RPS4 | 3 | **C**GG>**T**GG | R585W |
| 53 | RPS4 | 3 | **G**AA>**A**AA | E616K |
| 24 | RPS4 | 3 | C**C**A>C**T**A | P626L |
| 7 | RPS4 | 3 | TG**G**>TG**A** | W653* |
| 72 | RPS4 | 3 | **G**AG>**A**AG | E654K |
| 86 | RPS4 | 4 | T**G**C>T**A**C | C783Y |
| 90 | RPS4 | 4 | G**G**G>G**A**G | G828E |
| 12 | RPS4 | 4 | T**G**C>T**A**C | C887Y |
| 25 | RPS4 | 4 | **C**AG>**T**AG | Q911* |
| 41 | RPS4 | 4 | T**C**C>T**T**C | S914F |
| 21 | RPS4 | 4 | A**G**G>A**A**G | R938K |
| 60 | RPS4 | 4 | C**G**G>C**A**G | R947Q |
| 91 | RPS4 | 4 | G**G**G>G**A**G | G952E |
| 58 | RPS4 | 4 | G**G**G>G**A**G | G952E |
| 44 | RPS4 | 5 | G**G**G>G**A**G | G997E |
| 32 | RPS4 | 5 | G**G**G>G**A**G | G997E |
| 57 | RPS4 | 5 | **G**TG>**A**TG | V1109M |
| 70 | SID2/ICS1 | 8 | **C**CG>**T**CG | P403S |

1. Number of the sequenced *sushi* line [↑](#footnote-ref-1)
2. Candidate gene (CDS) sequenced [↑](#footnote-ref-2)
3. Localization of the mutation in the candidate gene CDS [↑](#footnote-ref-3)
4. Nucleotide mutation identified in the candidate gene codon [↑](#footnote-ref-4)
5. Resulting amino acid change in the candidate gene protein (* indicates STOP codon) [↑](#footnote-ref-5)
