# Supplementary material for: The Nuclear Immune Receptor RPS4 Is Required for RRS1SLH1-Dependent Constitutive Defense Activation in Arabidopsis thaliana
Source: PLoS Genet. 2014 Oct 23;10(10):e1004655. doi: 10.1371/journal.pgen.1004655 (PMC4207616; doi:10.1371/journal.pgen.1004655)
Supplement: Table S7 — Primers used in this study. (DOCX) [file pgen.1004655.s019.docx]

**Table S7.** Primers used in this study

| **Name** | **Sequence (5’🡪3’)** |
| --- | --- |
| RPS4_seq1_F | CCGACGATGCTTTTTCATTGGA |
| RPS4_seq1_R | TCATGACCTCCCTAAGTTCCA |
| RPS4_seq2_F | TCATCAATTTCCGTGGGGCA |
| RPS4_seq2_R | TATTAGGGCTGGGGCTCTGT |
| RPS4_seq3_F | ACAATCCTCACGTAGACAATCTCA |
| RPS4_seq3_R | TTAACCATTCACAAAAGCAATCAACAG |
| RPS4_seq4_F | TAAGCTACCATTGAAAGAAGTTCG |
| RPS4_seq4_R | CACGGCCTGGAATTTCCTCT |
| RPS4_seq5_F | TCAGCGGCTGCTCAACTTT |
| RPS4_seq5_R | AATCCCGGCAAGCTTCTTGT |
| RPS4_seq6_F | GTTCAGCACTTGCTTTCCTGG |
| RPS4_seq6_R | AAACTTGACAGGGCTCCCAC |
| RRS1_seq1_F | GAAAAAGCATCGTCGGCATAAACA |
| RRS1_seq1_R | AAGTGCGTCTCGTACACATCTC |
| RRS1_seq2_F | CCAGGTTTTCGCTTTATTCTACTC |
| RRS1_seq2_R | CTCATTTAAACCCTGGACCTCATAT |
| RRS1_seq3_F | TCTCGAGGGGTTTGACTGGCTAG |
| RRS1_seq3_R | TTCTGCGCCTCTCGATCTGT |
| RRS1_seq4_F | CCACATGTTGAAATTGATGTCCTTG |
| RRS1_seq4_R | GATCGTCCTCAACATCTCCAGGTTC |
| RRS1_seq5_F | GGTGGAACCAAGGTAAGCAA |
| RRS1_seq5_R | GCCACCAAGATATAACTGTTTCAGA |
| RRS1_seq6_F | CCAAACCATAGAGAGCTTGTGAATT |
| RRS1_seq6_R | TGGAAAGAAAGAGAGCTAATGGAGAGGT |
| RRS1_seq7_F | CGCTGACTTATGTAAAACACATACC |
| RRS1_seq7_R | CTTTGTCTGCTGATTGATAGGAAAG |
| RRS1_seq8_F | GTGATGCTACAGATGTTGGCA |
| RRS1_seq8_R | TGTAAACTTTAGTTCAATCTAATAAATTGCACGT |
| RRS1_seq9_F | GTTATATCGACGTTGGATGCAG |
| RRS1_seq9_R | CCAGCAAGTTTAGGATGATTACG |
| RRS1_seq10_F | GTGCGTAAGTGATCAAATTATGG |
| RRS1_seq10_R | TTGTCGAGATCACCATATGTTGG |
| EDS16_Seq1_F | AGCAATCTCTCTATACTACAAACACCA |
| EDS16_Seq1_R | GACCGAGTTCGAGTGTGTGT |
| EDS16_Seq2_F | GTCGAATTTTCCCGCGCAAT |
| EDS16_Seq2_R | CCACGAGCCAAAAACATCAGT |
| EDS16_Seq3_F | ACTGATGTTTTTGGCTCGTGG |
| EDS16_Seq3_R | TCGAAGAAATGAAGAGCTTGGAA |
| EDS16_Seq4_F | TCCAAGCTCTTCATTTCTTCGA |
| EDS16_Seq4_R | CCTGCCCTAGTTACAACCCG |
| EDS5_Seq1_F | CGAGGCGCCAAAAACAACAA |
| EDS5_Seq1_R | GACCAGTCCACCGGTTCAAA |
| EDS5_Seq2_F | TTGACTAGTCAACGCAAGGCT |
| EDS5_Seq2_R | TCAAGTGTTGGGCTTCATGA |
| EDS5_Seq3_F | TCATGAAGCCCAACACTTGA |
| EDS5_Seq3_R | AGAGACTTATTCAGCTGCTTGC |
| EDS5_Seq4_F | AACCAAAGGTTGTTTCTTGTTCAC |
| EDS5_Seq4_R | CCGTTTCTTGACATTGGTGCC |
| EF1alpha_qPCR_F | CAGGCTGATTGTGCTGTTCTTA |
| EF1alpha_qPCR_R | GTTGTATCCGACCTTCTTCAGG |
| PR1_qPCR_F | ATACACTCTGGTGGGCCTTACG |
| PR1_qPCR_R | TACACCTCACTTTGGCACATCC |
| PBS3_qPCR_F | TTCGCTGGCTTGTATAGGATGA |
| PBS3_qPCR_R | CTGGAAATGTTGAGGTGTCAGC |
| CBP60g_qPCR_F | GACATGACCTCAAGCTGGTCAC |
| CBP60g_qPCR_R | TGTGTGTCTCCGGACTTCCTTA |
| FMO1_qPCR_F | TGTGTTTGAAGATGGGACGACA |
| FMO1_qPCR_R | GTTCGAGCTGCTTTGGACGTAT |
| EDS5_qPCR_F | ACCTTTCTTCATGGCGTTGTCT |
| EDS5_qPCR_R | ATTGAAATCCGACGAGAACGA |
| NudT6_qPCR_F | TGGTCCAGGAGATTGATGGTC |
| NudT6_qPCR_R | CAAGAATGCTTGGTGGCTTTC |
| WRKY18_qPCR_F | GACAACCCGTCACCTAGAGCTT |
| WRKY18_qPCR_R | GTAGCATCCCCTTCAGAAGCAT |
| WRKY40_qPCR_F | CTTGTGCTCCAAGCTGTTCTGT |
| WRKY40_qPCR_R | GTTGAAGCTGAACCACCATGAG |
|  |  |
|  |  |
|  |  |
